# Evolution of P2A and P5A ATPases: ancient gene duplications and the red algal connection to green plants revisited

**DOI:** 10.1111/ppl.13008

**Published:** 2019-08-08

**Authors:** Michael Palmgren, Danny Mollerup Sørensen, Björn M. Hallström, Torbjörn Säll, Karin Broberg

**Affiliations:** ^1^ Department of Plant and Environmental Sciences University of Copenhagen Copenhagen Denmark; ^2^ Institute of Environmental Medicine Karolinska Institutet Stockholm Sweden; ^3^ Science for Life Laboratory KTH – Royal Institute of Technology Stockholm Sweden; ^4^ Department of Biology Lund University Lund Sweden

## Abstract

In a search for slowly evolving nuclear genes that may cast light on the deep evolution of plants, we carried out phylogenetic analyses of two well‐characterized subfamilies of P‐type pumps (P2A and P5A ATPases) from representative branches of the eukaryotic tree of life. Both P‐type ATPase genes were duplicated very early in eukaryotic evolution and before the divergence of the present eukaryotic supergroups. Synapomorphies identified in the sequences provide evidence that green plants and red algae are more distantly related than are green plants and eukaryotic supergroups in which secondary or tertiary plastids are common, such as several groups belonging to the clade that includes Stramenopiles, Alveolata, Rhizaria, Cryptophyta and Haptophyta (SAR). We propose that red algae branched off soon after the first photosynthesizing eukaryote had acquired a primary plastid, while in another lineage that led to SAR, the primary plastid was lost but, in some cases, regained as a secondary or tertiary plastid.

**Abbreviations**
BLAST: Basic Local Alignment Search Tool
CCT: chaperonin containing TCP‐1
eEF2: elongation factor 2
SPCA: secretory pathway Ca^2+^‐ATPases
TM: transmembrane segments

## Introduction

The evolutionary base of the eukaryotic phylogenetic tree has proven to be difficult to resolve. In particular it remains controversial on how green plants (Chloroplastida) evolved (Katz 2012). Chloroplastida, red algae (Rhodophyceae) and glaucophytes (Glaucophyta) all have primary plastids surrounded by a single membrane. Primary plastids evolved from an endosymbiotic relationship with a single cyanobacterial ancestor (Ponce‐Toledo et al. 2017, Sánchez‐Baracaldo et al. 2017). A common view is therefore that Chloroplastida, Rhodophyceae and Glaucophyta are united in a monophyletic group (Adl et al. 2019). However, although plastids are monophyletic, their eukaroytic hosts need not be monophyletic (reviewed by Mackiewicz and Gagat 2014), and multiple recent phylogenomic studies involving nuclear markers have failed to conclude that Chloroplastida, Rhodophyceae and Glaucophyta form a monophyletic clade excluding all other eukaryotic supergroups (Brown et al. 2013, Cavalier‐Smith et al. 2014, Yabuki et al. 2014, Burki et al. 2016).

The state‐of‐the‐art approach to understanding the pattern of evolution across the tree of life is to analyze genome‐level data. This approach of phylogenomics, to resolve problematic branches in phylogenetic trees, is based on increasing the number of genes analyzed at a genomic scale, in which hundreds to thousands of loci are examined. Generation of such massive datasets is presumed to dampen out phylogenetic biases and artifacts that may impact single gene phylogenies (Delsuc et al. 2005). Phylogenomic approaches often depend on building datasets of concatenated orthologs. Such studies require assumptions of orthology among sequences and exclude paralogs (Dessimoz et al. 2006). Orthologs are genes derived from a single ancestral gene in the last common ancestor of the compared species, whereas paralogs are genes related through duplication (Koonin 2005). Thus, per definition, building datasets of concatenated orthologs eliminates phylogenetic information resulting from gene duplication and gene loss events (Katz 2012). The use of concatenated sequences may therefore be problematic when investigating ancient evolutionary events, where traces of similarity, e.g. from gene duplications, can have eroded with time. Analyses of many genetic markers are also vulnerable to systematic errors that exacerbate any existing problems with the data, which can lead to phylogenetic reconstructions that reflect false evolutionary relationships but with high statistical support (Philippe et al. 2011, Philippe and Roure 2011). Therefore, there is a need for complementary methods for assigning evolutionary relationships at deep branches.

As a supplementary approach to phylogeny, Baldauf and Palmer (1993) used the identification of rare mutational events, also called synapomorphies, which are molecular signatures in the proteins analyzed, to demonstrate the sister‐group relationship of fungi and animals. In the fungal and animal sequences analyzed, a 12‐amino acid insertion in translation elongation factor 1 alpha and three small gaps in enolase were identified, and all four insertions/deletions were found to be uniquely shared by animals and fungi relative to plants, protists and bacteria (Baldauf and Palmer 1993). Still, analysis of synapomorphies is not often used in evolutionary studies. It is more cumbersome and delicate than traditional phylogenomics, because it requires extensive knowledge of the proteins analyzed. Consequently, most broad‐scale phylogenetic analyses of eukaryotes rely purely on computational approaches, and there is little or no consideration of the significance of evolutionary changes at the molecular or biochemical levels.

A common approach in phylogeny is to study well‐characterized families of proteins that are conserved and present in all life forms. One such family that so far has been overlooked in evolutionary studies is the P‐type ATPase family. P‐type ATPases constitute a ubiquitous family of cation pumps, named because they use ATP as their energy source and form a phosphorylated (hence P‐type) reaction cycle intermediate (Palmgren and Nissen 2011). The P‐type ATPase superfamily is divided into five classes of membrane‐bound pumps, P1–P5, which are further divided into subfamilies (Axelsen and Palmgren 1998). The sarco/endoplasmic reticulum Ca^2+^ ATPase (SERCA, P2A) and the P5A subfamilies are highly conserved and widely distributed, making them suitable for evolutionary studies.

In a search for slowly evolving nuclear genes that may cast light on the deep evolution of plants, we carried out phylogenetic analyses and identified synapomorphies within two well‐characterized subfamilies of P‐type pumps (P2A and P5A ATPases) from representative branches of the eukaryotic tree of life. Both genes were found to be duplicated before the diversification of eukaryotic supergroups. Furthermore, sequences from Chloroplastida showed evidence of a closer relationship to Cryptophyta, Haptophyta and Stramenopiles than to Rhodophyta.

## Materials and methods

### Defining criteria for selecting P‐type ATPases for analysis

Sarcoplasmic/endoplasmic Ca^2+^ ATPase (SERCA) pumps (P2A ATPases) are ubiquitous and the best‐characterized P‐type ATPases. Almost all residues in this pump have been mutagenized and functionally characterized (Møller et al. 2010). A characteristic of SERCA pumps is the presence of two Ca^2+^ ion binding sites, sites 1 and 2, that are coordinated by amino acid residues in transmembrane (TM) helices TM4, TM5, TM6, and TM8 (Møller et al. 2010; Fig. S1). P5A ATPases represent a conserved subfamily of P‐type ATPases that are present in the endoplasmic reticulum and are expected to serve an important function, as their deletion causes severe endoplasmic reticulum stress. However, their biochemical ligand has not been identified yet (Sorensen et al. 2015). P5A pumps are easily recognizable due to specific sequence motifs in most transmembrane domains (Sorensen et al. 2015), including a PQ.L motif in TM1 (Fig. S2). P5A pumps are interesting from an evolutionary perspective as they are completely absent from prokaryotes, but ubiquitous in eukaryotes (Møller et al. 2008). They are thus likely to have appeared with the first eukaryote.

### Identification of P‐type ATPase sequences

The strategy used to identify P‐type ATPase sequences was as previously reported (Palmgren et al. 2017). Sequences of SERCA2‐like calcium pumps and Spf1p‐like P5A ATPases were identified in the NCBI protein database using the Basic Local Alignment Search Tool (BLAST) program (http://blast.ncbi.nlm.nih.gov/) and used to search the genomes of 153 species representing major eukaryotic phyla and prokaryotes. For each species, BLAST searches were carried out using human SERCA2 (P‐type P2A pump, ATP2A2; P16615) or *S. cerevisiae* Spf1p (P‐type P5A pump, P39986) amino acid sequences. Additional searches for P‐type ATPase homologs were carried out through a BLAST search at the Joint Genome Institute (JGI) Genome Portal (http://genome.jgi.doe.gov/), the PlantGDB database (http://www.plantgdb.org/PpGDB/cgi-bin/blastGDB.pl#PPpep:Pp1s6_11V6.1), the *Porphyridium purpureum* Genome Project server (http://cyanophora.rutgers.edu/porphyridium/), the Phytozome Plant Genomics Resource (https://phytozome.jgi.doe.gov/pz/portal.html#!search?show=BLAST), the Conifer Genome Network (CGN) Dendrome Database (http://dendrome.ucdavis.edu/resources/blast/), the Mnemiopsis Genome Project Portal (http://dendrome.ucdavis.edu/resources/blast/), the Cyanophora Genome Project server (http://cyanophora.rutgers.edu/cyanophora/blast.php), and the Plantmorphogenesis server (http://www.plantmorphogenesis.bio.titech.ac.jp/~algae_genome_project/klebsormidium/klebsormidium_blast.html). All sequences identified from Rhodophyceae, Chlorophyta, Stramenopiles, Alveolata, Haptophyta, Cryptophyta, and Rhizaria were included in the analysis.

All hits in each species with significant similarity to the query (expected value of <e^−30^) were selected and their relationship to each P‐type ATPase subfamily was investigated by constructing phylogenetic trees for all candidate sequences in each individual genome together with a set of known P‐type ATPases using MUSCLE alignment (Edgar 2004). Maximum likelihood phylogeny reconstruction was then performed in a Gamma distributed LG model (Le and Gascuel 1993) and implemented in MEGA6 (Tamura et al. 2013). The nature of the individual sequences was subsequently confirmed by manual inspection for conserved sequence motifs characteristic of P‐type P2A SERCA (Møller et al. 2010) and P5A (Møller et al. 2008) pumps. For example, Ca^2+^ binding site 2 (Fig. S1) is not only present in SERCA but also in those Ca^2+^ pumps that only have a single Ca^2+^ binding site, namely the P2A secretory pathway Ca^2+^‐ATPases (SPCA) and the P2B plasma membrane Ca^2+^‐ATPases. However, Ca^2+^ binding site 1 is specific for SERCA pumps and is generated by residues in TM5, TM6 and TM8 (Fig. S1; Møller et al. 2010). Sequences that did not fulfill the above criteria, and are therefore not likely to be P2A or P5A ATPases, were eliminated from the dataset. As many genomes in the databases are still in draft form, the predicted P‐type ATPases often did not represent complete proteins, and partial sequences lacking conserved P‐type ATPase motifs were removed following alignment. The resulting data set contained SERCA2‐like P2A calcium pumps and Spf1p‐like P5A ATPases from 118 and 97 species, respectively. Accession numbers are listed in Table S1 (P2A ATPases) and Table S2 (P5A ATPases). For each protein in Eubacteria, the chromosomal positions of genes corresponding to the investigated proteins were compared to judge whether or not they were genetically linked.

First, using a combination of the general P‐type ATPase sequence motifs (Axelsen and Palmgren 1998) and the specific Ca^2+^ binding site residues (Fig. S1) as search motifs, SERCA pumps were identified in eukaryotic genomes (Table S1). No SERCA pumps were identified in the genomes of organisms belonging to the Archaea or to the eukaryotic Amoebozoa and Aposozoa. Several isoforms of SERCA pumps were present in mammals (e.g. three in *Homo sapiens*, Altshuler et al. 2012) and land plants (e.g. four in *A. thaliana*; Evans and Williams 1998, Altshuler et al. 2012). Adding to this list, we identified up to three isoforms in many genomes of the protozoal supergroups Stramenopiles, Alveolata and Rhizaria (Table S1). In contrast to other P‐type ATPase subfamilies, P5A ATPases have previously been reported to typically only be present in single copies (Sorensen et al. 2015). However, in this analysis, two copies were identified in most Stramenopiles and in the Alveolata *Perkinsus marinus* (Table S2).

### Phylogenetic analysis of P‐type ATPases

Protein sequence alignment was performed using MUSCLE (Edgar 2004) implemented in MEGA6. Positions due to insertions in fewer than 50% of the sequences were discarded and ambiguous data following manual inspection were eliminated because it cannot be assumed that sequences found in ambiguously aligned regions in different taxa are homologous, and any bias in the method of sequence alignment may influence the result (Baldauf et al. 1996). This resulted in a total of 673 (177 amino acid sequences) and 1058 (111) amino acid residue positions in the final dataset of P2A and P5A pumps, respectively. The evolutionary history was inferred assuming an LG (Le and Gascuel 1993) +INVGAMMA model, as identified by ProtTest (Abascal et al. 2005). Phylogenetic analyses were subsequently conducted using Bayesian inference and maximum likelihood methods. Bayesian inference was performed with MrBayes 3.2.6 (Ronquist et al. 2012) and maximum likelihood analyses with RAxML 8.2.9 (Stamatakis 2014) and, in initial analyses, MEGA6 (Tamura et al. 2013). In the RAxML analyses, clade robustness was assessed with 1000 rapid bootstrap inferences followed by thorough analysis of maximum likelihood to obtain statistical support for the placement of nodes. The MrBayes analyses were performed using the following settings: eight chains of Markov chain Monte‐Carlo iterations and a heated parameter of 0.05 with trees sampled every 1000 generations. The average standard deviations of split frequencies at termination of the analyses after 1 000 000 generations were 0.005606 for the P2A tree and 0.003138 for the P5A tree. Both the MrBayes and RAxML analyses were run on the CIPRES Science Gateway (Miller et al. 2010) in the Extreme Science and Engineering Discovery Environment (XSEDE). Sequence synapomorphies specific for the clades identified were detected by manual inspection of protein sequences in each clade.

### Comparison of unconstrained vs constrained trees

In constrained trees, sequences belonging to selected supergroups were forced to cluster monophyletically (Shimodaira and Hasegawa 1999). For comparisons of constrained vs unconstrained trees, RAxML was used. All RAxML analyses were performed using the same model as used for MrBayes (PROTGAMMILGF). First, the branch lengths of the MrBayes tree were re‐calculated in RAxML using the same model as for MrBayes, and the ‘optimize model parameters + branch lengths for given input tree’ (−f e) mode in RAxML. An unconstrained RAxML tree was also created. Second, two different constrained trees were determined by providing RAxML with multifurcating constraint trees (using the ± g option). Finally, the MrBayes tree (with recalculated branch lengths) was compared with the unconstrained tree.

### Identification of EF2 sequences

Amino acid sequences for elongation factor 2 (EF2) were retrieved from sequence databases using BlastP with an *E*‐value cutoff of e^−30^. The identity of sequences was confirmed by comparing them with conserved sequence motifs from EF2 (Atkinson and Baldauf 2010). Accession numbers are listed in Table S3.

## Results

### Phylogenetic analysis of P‐type ATPase subfamilies

**Figure 1:**
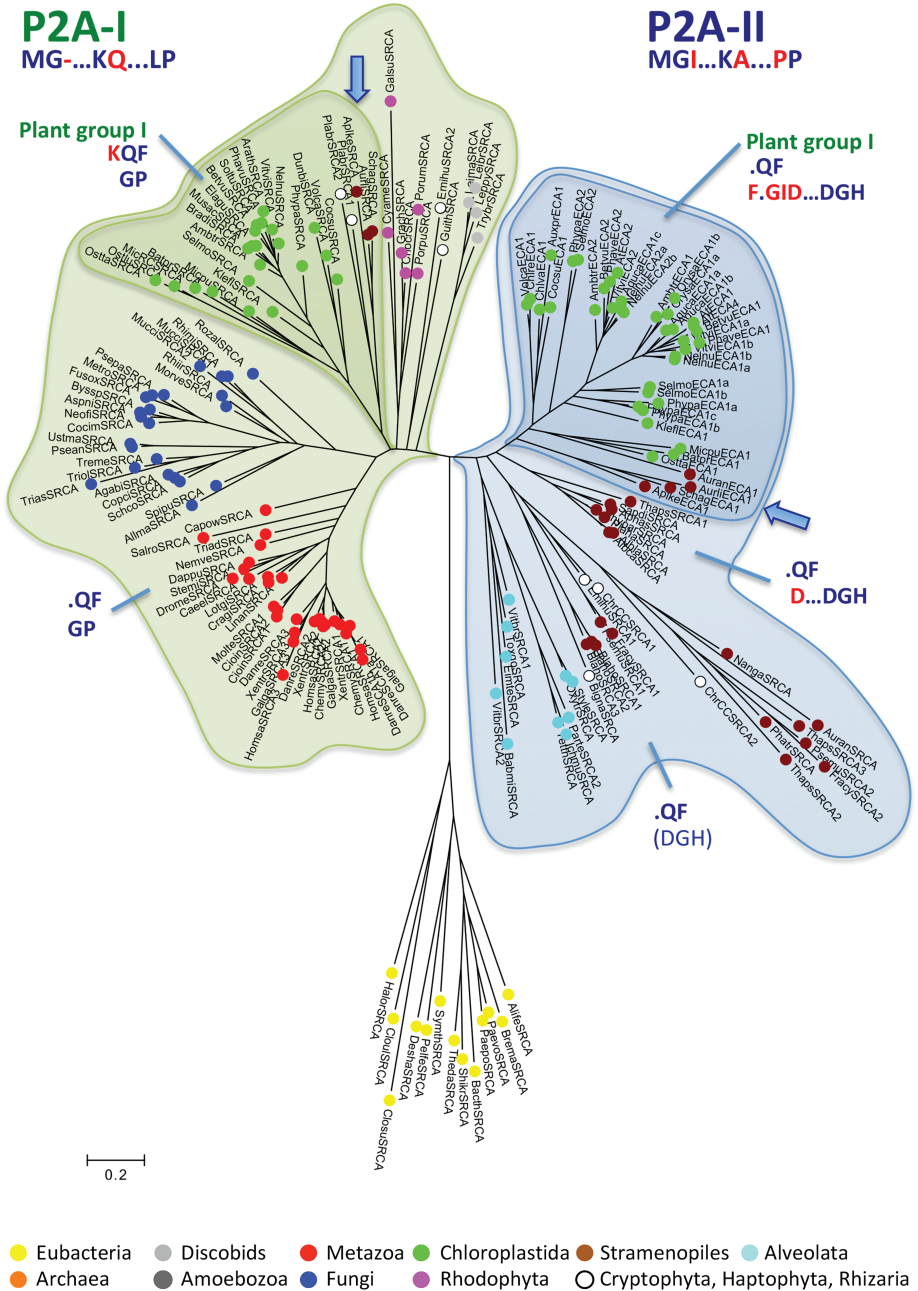
Phylogenetic analysis of P2A SERCA‐like proteins reveals a gene duplication event at the time of the last common eukaryotic ancestor. Two major branches are identified (P2A‐I and P2A‐II; marked by different color shading), each characterized by its own synapomorphy indicated below each branch: P2A‐I: A deletion of a single residue in the phosphorylation (P) domain; P2A‐II: A double pro motif in transmembrane segment 6 (TM6). The tree is the result of a maximum likelihood analysis using RAxML and involving 177 amino acid sequences from 118 species. The best tree (likelihood −99 967.250889) after 1000 bootstrap rounds is shown, as described in section Methods. There were a total of 673 positions in the final dataset. The tree was rooted with eubacterial sequences (from Firmicutes). Scale bar, 0.2 amino acid substitutions per site. Abbreviated sequence names are given in full in Table S1. Each sequence in the tree is marked with a dot colored according to the taxonomic supergroup to which it belongs. Color codes are given below the figure.

**Figure 2:**
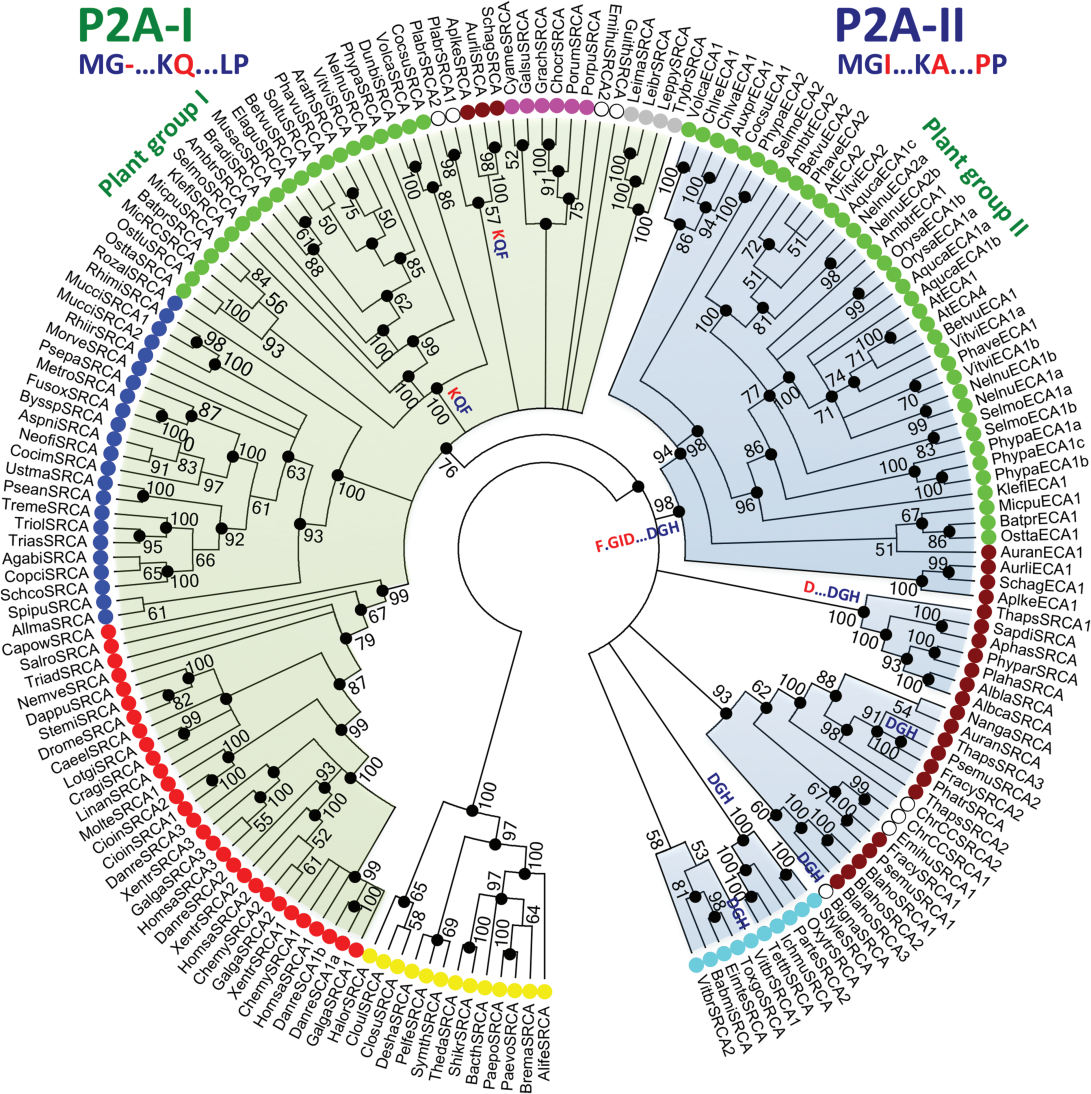
Evolutionary relationship between clades of P2A ATPases. A bootstrap consensus tree was generated from the tree shown in Fig. 1 in which branches corresponding to partitions reproduced in fewer than 50% of the 1000 bootstrap replicates were collapsed. A separate Bayesian inference analysis was carried out using the program MrBayes, which resulted in a tree similar to that shown in Fig. 1. Black dots at nodes in the RAxML consensus tree indicate maximum statistical support (*P* = 1) in the Bayesian inference analysis. The Bayesian interference analysis was run for 1 000 000 generations and the average standard deviation of split frequencies between the resulting trees was 0.005606. Identified synapomorphies are given at the base of major clades. Abbreviated sequence names are given in full in Table S1. Color codes are given in the legend to Fig. 1.

**Figure 3:**
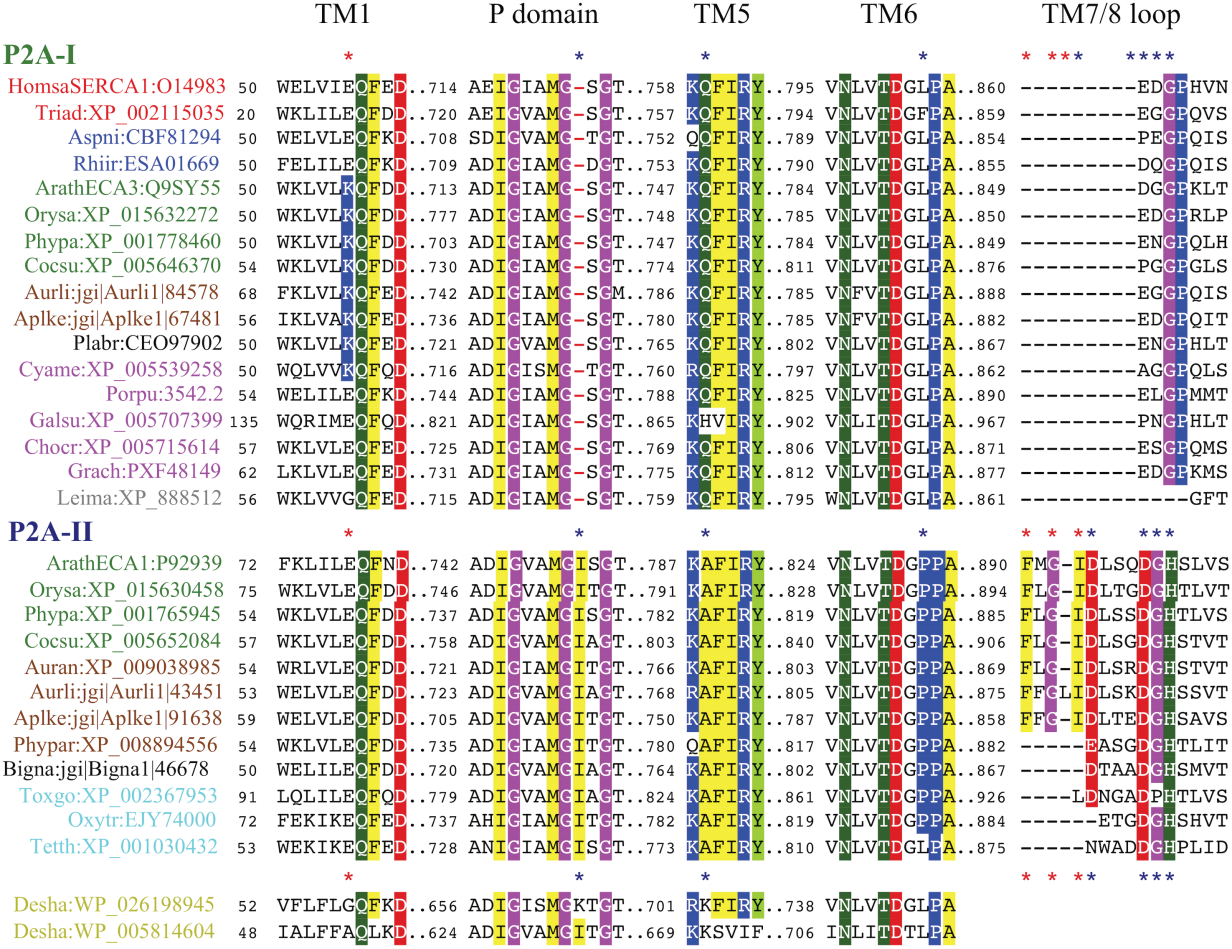
Synapomorphies in catalytic domains and transmembrane segments of P2A SERCA‐like ATPases define two groups of eukaryotic P2A ATPases. One group of sequences (P2A‐I) carries a single amino acid residue deletion in the P domain and another group (P2A‐II) a PP motif in TM6. Chloroplastida and Stramenopiles (here represented with sequences from non‐photosynthetic organisms) are represented in both groups. Some sequences in Alveolata that lack the deletion in the P domain also lack the PP motif. Residues that are conserved in all species and those that represent synapomorphies are colored. Sequences are from selected organisms (abbreviated names are in parentheses): Animals (red text), *Homo sapiens* (Homsa) and *Trichoplax adhaerens* (triad); fungi (blue text), *Aspergillus niger* (Aspni) and *Rhizophagus irregularis* (Rhiir); Chloroplastida (green text), *Arabidopsis thaliana* (Arath), *Oryza sativa* (Orysa), *Physcomitrella patens* (Phypa) and *Coccomyxa subellipsoidea* (Cocsu); Stramenopiles (brown text), *Aureococcus anophagefferens* (Auran), *Aurantiochytrium limacinum* (Aurli), *Aplanochytrium kerguelense* (Aplke), and *Phytophthora parasitica* (Phypar); Rhizaria (black text), *Plasmodiophora brassicae* (Plabr) and *Bigelowiella natans* (Bigna); Rhodophyceae (cyan text), *Cyanidioschyzon merolae* (Cyame); *Porphyridium purpureum* (Porpu); *Galdieria sulphuraria* (Galsu); *Chondrus crispus* (Chocr); *Gracilariopsis chorda* (Grach); Alveolata (turquoise text), *Toxoplasma gondii* (Toxgo), *Oxytricha trifallax* (Oxytr) and *Tetrahymena thermophila* (Tetth); Discobids (light gray text), *Leishmania major* (Leima); and eubacteria (yellow text), *Desulfitobacterium hafniense* (Desha). Asterisks indicate the position of synapomorphies. Blue asterisks indicate synapomorphies common for major clades. Red asterisks indicate synapomorphies common for sub‐clades discussed in the text.

**Figure 4:**
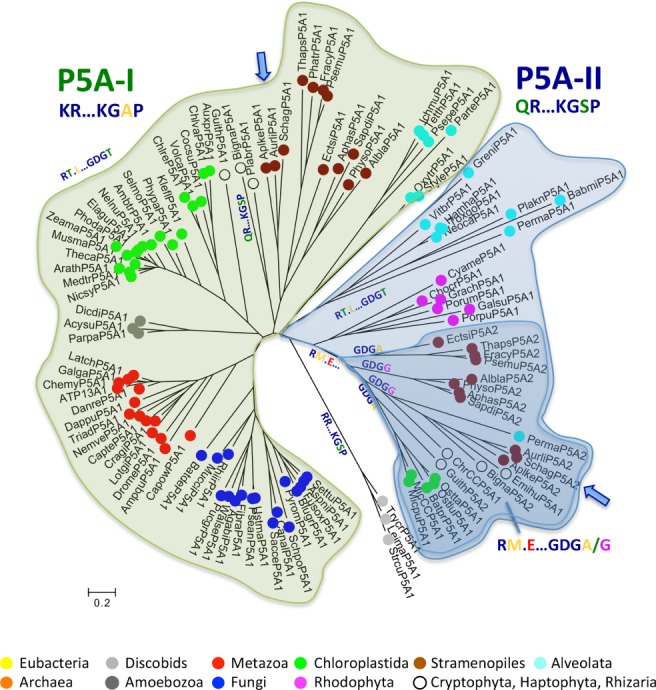
Phylogenetic analysis of P5A‐like proteins reveals a gene duplication event before eukaryotes diversified into supergroups. Two major branches are identified (P5A‐I and P5A‐II; marked by different color shading), each of which is characterized by two synapomorphies indicated below each branch: P5A‐I: KR…KGAP indicates a Lys‐Arg (KR) motif and a Lys‐Gly‐Ala‐Pro (KGAP) motif in the N domain; P5A‐II: QR…KGSP indicates a Gln‐Arg (QR) motif and a Lys‐Gly‐Ser‐Pro (KGSR) motif in the N domain. Each sequence in the tree is marked with a dot colored according to which taxonomic supergroup it belongs to. Color codes are given in Fig. 1. The tree is the result of a maximum likelihood analysis using RAxML and involving 111 amino acid sequences from 97 species. Shown is the best tree (likelihood – 149 464.799485) after 1000 bootstrap rounds, as described in section Methods. There were a total of 1058 positions in the final dataset. Scale bar, 0.2 amino acid substitutions per site. Abbreviated sequence names are given in full in Table S2. Color codes are given in the legend to Fig. 1.

**Figure 5:**
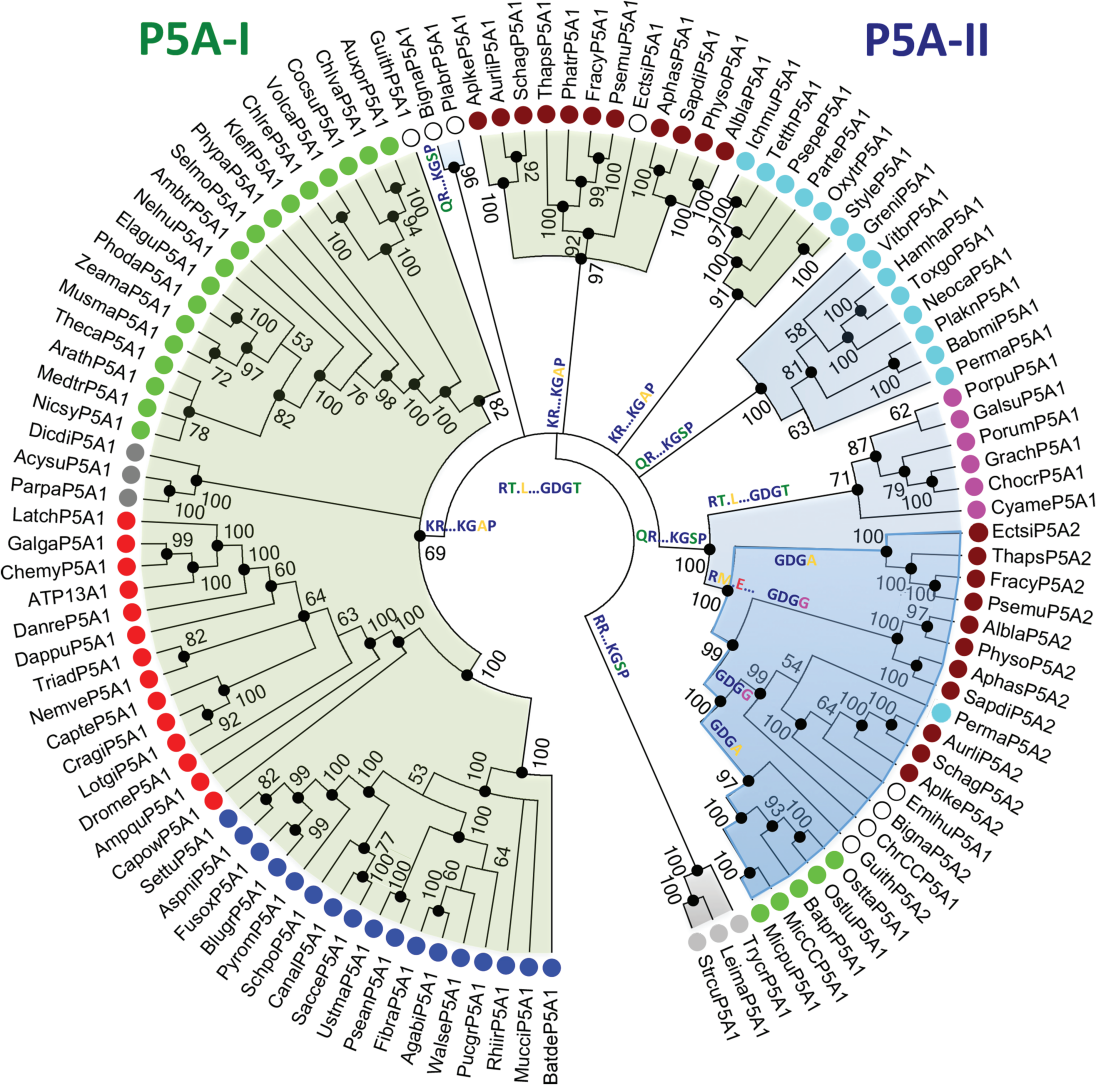
Evolutionary relationship between clades of P5A ATPases. A bootstrap consensus tree was generated in which branches corresponding to partitions reproduced in fewer than 50% of the 1000 bootstrap replicates were collapsed. A separate Bayesian inference analysis was carried out using the program MrBayes, which resulted in a tree similar to that shown in Fig. 3. Black dots at nodes in the RAxML consensus tree indicate maximum statistical support (*P* = 1) in the Bayesian inference analysis. The Bayesian interference analysis was run for 1 000 000 generations and the average standard deviation of split frequencies between the resulting trees was 0.003138. Defining synapomorphies are given at the base of major clades. Abbreviated sequence names are given in full in Table S2. Color codes are given in the legend to Fig. 1.

**Figure 6:**
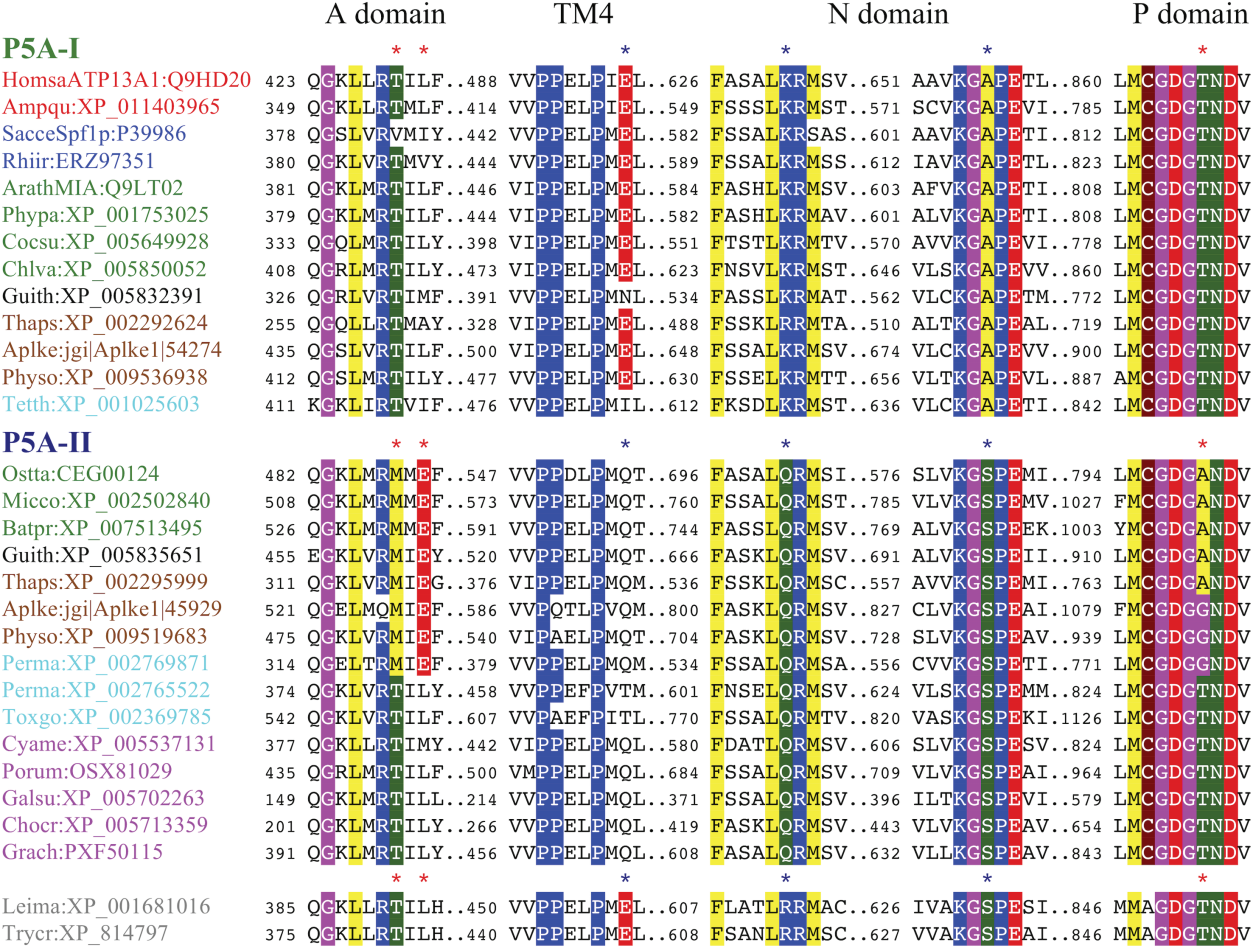
Synapomorphies in catalytic domains and transmembrane segments of P5A ATPase‐like proteins define two groups of eukaryotic pumps. One group of sequences (P5A‐I: ‘KR…KGAP’) has KR and KGAP motifs in the N domain, whereas another group (P5A‐II: ‘QR…KGSP’) has QR and KGSP motifs. Chloroplastida, Stramenopiles and Alveolata are represented in both groups. Residues that are conserved in all species and those that represent synapomorphies are colored. Sequences are from selected organisms (abbreviated names are in parentheses): animals (red text), *Homo sapiens* (Homsa) and *Amphimedon queenslandica* (Ampqu); fungi (blue text), *Saccharomyces cerevisiae* (Sacce) and *Rhizophagus irregularis* (Rhiir); Chloroplastida (green text), *Arabidopsis thaliana* (Arath), *Physcomitrella patens* (Phypa), *Coccomyxa subellipsoidea* (Cocsu), *Chlorella variabilis* (Chlva), *Ostreococcus tauri* (Ostta), *Micromonas commoda* (Micco) and *Bathycoccus prasinos* (Batpr); Cryptophyta (black text), *Guillardia theta* (Guith); Stramenopiles (brown text), *Thalassiosira pseudonana* (Thaps), *Aplanochytrium kerguelense* (Aplke) and *Phytophthora sojae* (Physo); Alveolata (turquoise text), *Tetrahymena thermophila* (Tetth), *Perkinsus marinus* (Perma) and *Toxoplasma gondii* (Toxgo); Rhodophyceae (cyan text), *Cyanidioschyzon merolae* (Cyame), *Porphyra umbilicalis* (Porum); *Galdieria sulphuraria* (Galsu); *Chondrus crispus* (Chocr); *Gracilariopsis chorda* (Grach); and Discobids (light gray text), *Leishmania major* (Leima) and *Trypanosoma cruzi* (Trycr). Asterisks indicate the position of synapomorphies. Blue asterisks show synapomorphies common for major clades. Red asterisks show synapomorphies common for sub‐clades discussed in the text.

Phylogenetic analyses were carried out for Ca^2+^ ATPase (SERCA) pumps (P2A ATPases; Figs 1, 2, 3) and P5A ATPases (Figs 4, 5, 6). We analyzed all isoforms identified in the genomes of representative eukaryotic species after having confirmed by sequence analysis that they contain the signature motifs essential for the biochemical function of proteins in each group. Definition and identification information is provided in section Materials and Methods. Maximum likelihood and Bayesian inference analyses yielded identical tree topologies for both P2A and P5A ATPases (Figs 2 and 4). Both P‐type ATPase trees appeared complex and contained in several instances more than one clade of each eukaryotic supergroup but at different positions in the tree (Figs. 1 and 3). In several respects the major clades mirrored each other. The presence of two mirrored clades was particularly evident in P2A ATPases (Figs. 1 and 2). Notably, this feature is absent in a phylogenetic tree of P2A ATPases based on orthologous sequences only (Palmgren et al. 2017). To examine the robustness of our findings, we first evaluated constrained vs unconstrained P2A and P5A trees constructed by RAxML, followed by a Shimodaira‐Hasegawa test. We evaluated the effects of forcing all Chloroplastida and Stramenopiles, respectively, to form monophyletic clusters in constrained trees. We compared the consensus MrBayes tree with the constrained and unconstrained trees constructed using RAxML and found that the constrained trees resulted in significantly inaccurate (i.e. lower likelihood) trees compared to the unconstrained trees for both Chloroplastida and Stramenopiles (Table 1). One interpretation of these findings is that there are two basal clades of eukaryotic P2A and P5A pumps.

**Table 1:** Shimodaira–Hasegawa (SH) test (Shimodaira and Hasegawa 1999) to determine whether the data support our conclusion that P‐type ATPase duplications occurred before the divergence of major supergroups, using topological constraints that enforce the monophyly of the supergroups. The SH test results indicate that the topological constraints imposed to ignore such a split are significantly worse (*P* < 0.01) than the unconstrained maximum likelihood topology. Abbreviations: ML, maximum likelihood; D(LH), difference in likelihood scores between the two trees; sd, sd of D(LH). ^a^Tree presented in Fig. 1; ^b^Tree in Fig. 4.

	ML	D(LH)	SD	*P*
P2A ATPases^a^				
Best tree	−99 967.250886			
Constraint: Land plants	−100 388.245068	−420.994182	39.540803	<0.01
Constraint: Chloroplastida	−100 204.634459	−237.383573	46.337588	<0.01
Constraint: Stramenopiles	−100 428.728484	−461.477598	54.938916	<0.01
P5A ATPases^b^				
Best tree	−149 464.799572			
Constraint: Chloroplastida	−149 956.576393	−491.776821	52.049906	<0.01
Constraint: Stramenopiles	−150 699.582335	−1234.782764	71.623601	<0.01
Constraint: Alveolata	−149 717.706215	−252.906644	37.282026	<0.01

### Synapomorphies in P‐type ATPases linking eukaryotic supergroups to each other

To obtain further information that could help interpret the phylogenetic trees, we carried out a detailed protein sequence analysis to identify synapomorphies specific or common to the various eukaryotic clades.

***P2A ATPases** –* Synapomorphies were found to be characteristic for each of the two major clades of P2A ATPases identified in the phylogenetic analysis above. Sequences in one of the major clades (named P2A‐I) were found to carry a one‐amino acid residue deletion in a conserved P‐type ATPase segment (Axelsen and Palmgren 1998) in the phosphorylation (P) domain, a conserved glutamate (Q) in TM5 and a single conserved proline in TM6 (P in single letter code; labeled MG‐…KQ…LP in Figs 1 and 2; marked with black asterisks in Fig. 6; Table S1). In the second major clade (named P2A‐II) sequences did not have the above‐mentioned deletion in the P domain, had a conserved alanine (A) in TM5, and had a double proline (PP) motif in TM6 (labeled MGI…KA…PP in Figs 1 and 2; marked with black asterisks in Fig. 3; Table S1).

Monophyletic clades of green plants (named Plant Group I and II) were present in both the P2A‐I and P2A‐II clades, as were representatives of Stramenopiles, Alveolata and Rhizaria (Fig. 1). Metazoan (animal) and fungal P2A pumps were only present in the P2A‐I clade. Plant Group II in the P2A‐I clade had a common root with sequences from Stramenopiles (Bayesian inference value: 1; bootstrap value: 98), whereas Plant Group I in the P2A‐II clade had a common root with sequences from the protozoan supergroups Stramenopiles and Rhizaria (Bayesian inference value: 0.99; the bootstrap value was insignificant). Other protozoal supergroups such as Rhizaria, Alveolata, Haptophyta and Cryptophyta also appeared to be present in the P2A‐I clade, but the exact placement of their roots received little statistical support. Notably, representatives of the non‐photosynthetic Stramenopilean group Labyrinthulomycetes (indicated by blue arrows in Fig. 1) grouped with Chloroplastida in both major clades. In the P2A‐I clade, this relationship was confirmed by the identification of a synapomorphy in TM1, where a positively charged lysine (K; marked by a red asterisk in Fig. 3) preceded a conserved Gln‐Phe (QF) motif, which united Chloroplastida, Stramenopiles, Cryptophyta and Rhizaria to the exclusion of other eukaryotic supergroups including Rhodophyceae and Alveolata. In the P2A‐II clade, a relationship between Chloroplastida and sequences in Stramenopiles was confirmed by identification of a synapomorphy in the extracytoplasmic loop between TM7 and TM8, where a Phe‐x‐Gly‐Ile motif (F.GI; marked by a red asterisk in Fig. 3) united these groups.

Taken together, the synapomorphies in P2A ATPases provided evidence of two monophyletic clades of eukaryotic P2A ATPases. The two clades may represent an ancient sub‐functionalization of these Ca^2+^ ATPases that has yet to be characterized. In addition, this analysis suggests that these pumps originated from a gene duplication event in the early history of eukaryotes, i.e. before the split into the supergroups known today. In fungi and animals, one of the two copies was lost after the duplication event, but the remaining copy was duplicated later in evolution. In other supergroups, both copies were retained throughout evolution and both eventually underwent additional duplications. Further, the analysis revealed a link between Chloroplastida and Stramenopiles, which may suggest an evolutionary relationship between these two lineages.

***P5A ATPases** –* Based on synapomorphies, the sequences in the tree could be separated into two distinct groups. One group (named P5A‐I; labeled ‘KR…KGAP’ in Figs 4 and 5) was characterized by a Glu (E) in TM4 (marked by a black asterisk in Fig. 6) and two conserved KR and KGAPE motifs in the nucleotide binding N domain (Fig. 6). The other group (named P5A‐II; labeled ‘QR…KGSP’ in Figs 4 and 5) had no Glu in TM4 and QR and KGSPE motifs in the N domain (Fig. 6).

One Stramenopiles clade was placed with the land plant clade in the P5A‐I group and the other Stramenopiles clade was placed with a clade of green algae (including sequences from Mamiellophyceae) in the P5A‐II group. Constrained trees, where sequences of Chloroplastida and Stramenopiles, respectively, were forced together in monophyletic clades, had significantly poorer statistical support than the unconstrained trees (Table 1).

Within the P5A‐II group, a robust clade with 100% bootstrap support linked Chloroplastida (green algae herein) to Stramenopiles (including Labyrinthulomycetes), Cryptophyta, Haptophyta, Rhizaria, and a single alveolate sequence (from *P. marinus*), but to the exclusion of Rhodophyceae. This clade (marked ‘RM.E…GDGA/G’ in Figs 4 and 5) was characterized by two synapomorphies. First, a conserved Glu (E) was present in the A domain at a position where all other sequences had a hydrophobic residue (Fig. 6). Second, in this group, the Thr (T) residue in the conserved ‘GDGTND’ motif of the P domain was replaced with either an Ala (A) or Gly (G) residue (Fig. 6). Stramenopiles could have either A or G at this position, but only G was observed in green algae (Fig. 6; Table S2).

Taken together, although statistically significant values at the root of the tree could not be obtained by maximum likelihood or Bayesian inference statistics, the analysis of P5A ATPases synapomorphies provide strong evidence that there are two distinct monophyletic groups in the tree, and that both major clades separated before the origin of the eukaryotic supergroups of today.

### Evolution of ancient proteins involved in protein synthesis

**Figure 7:**
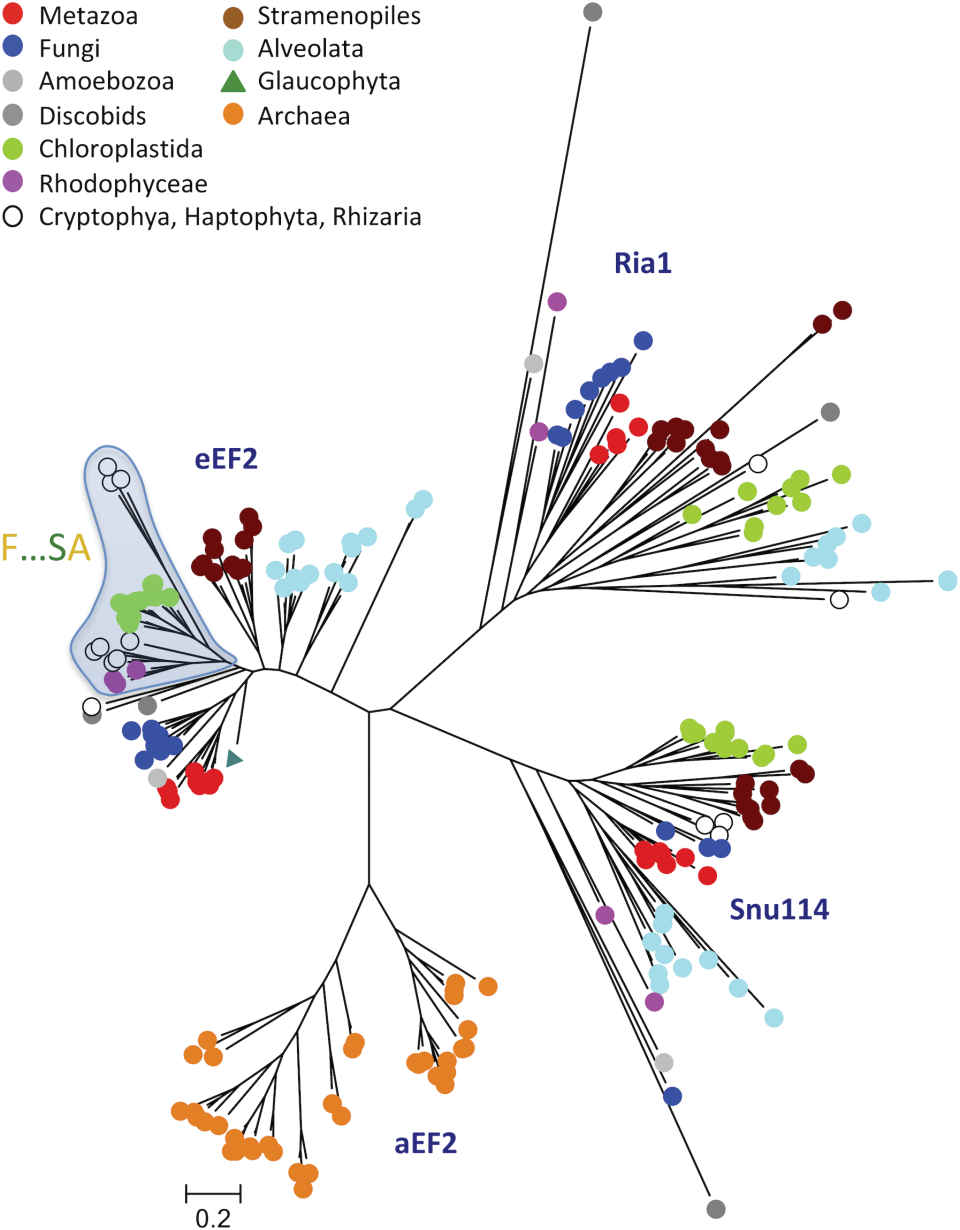
Phylogenetic tree depicting the evolutionary history of elongation factor 2 (EF2). Each sequence in the tree is marked with a dot colored according to the taxonomic supergroup to which it belongs. A group of sequences in the eEF2 clade is marked in which sequences share a number of common synapomorphies (shown in Fig. 9; here abbreviated as F…SA). The tree is the result of a maximum likelihood analysis using RAxML and involves 202 amino acid sequences from 110 species. Shown is the best tree (likelihood −158 581.149729) after 1000 bootstrap rounds, as described in section Methods. There were a total of 801 positions in the final dataset. As EF2 is derived from Archaea (Atkinson 2015), the tree was rooted with archaeal sequences. Scale bar, 0.2 amino acid substitutions per site.

**Figure 8:**
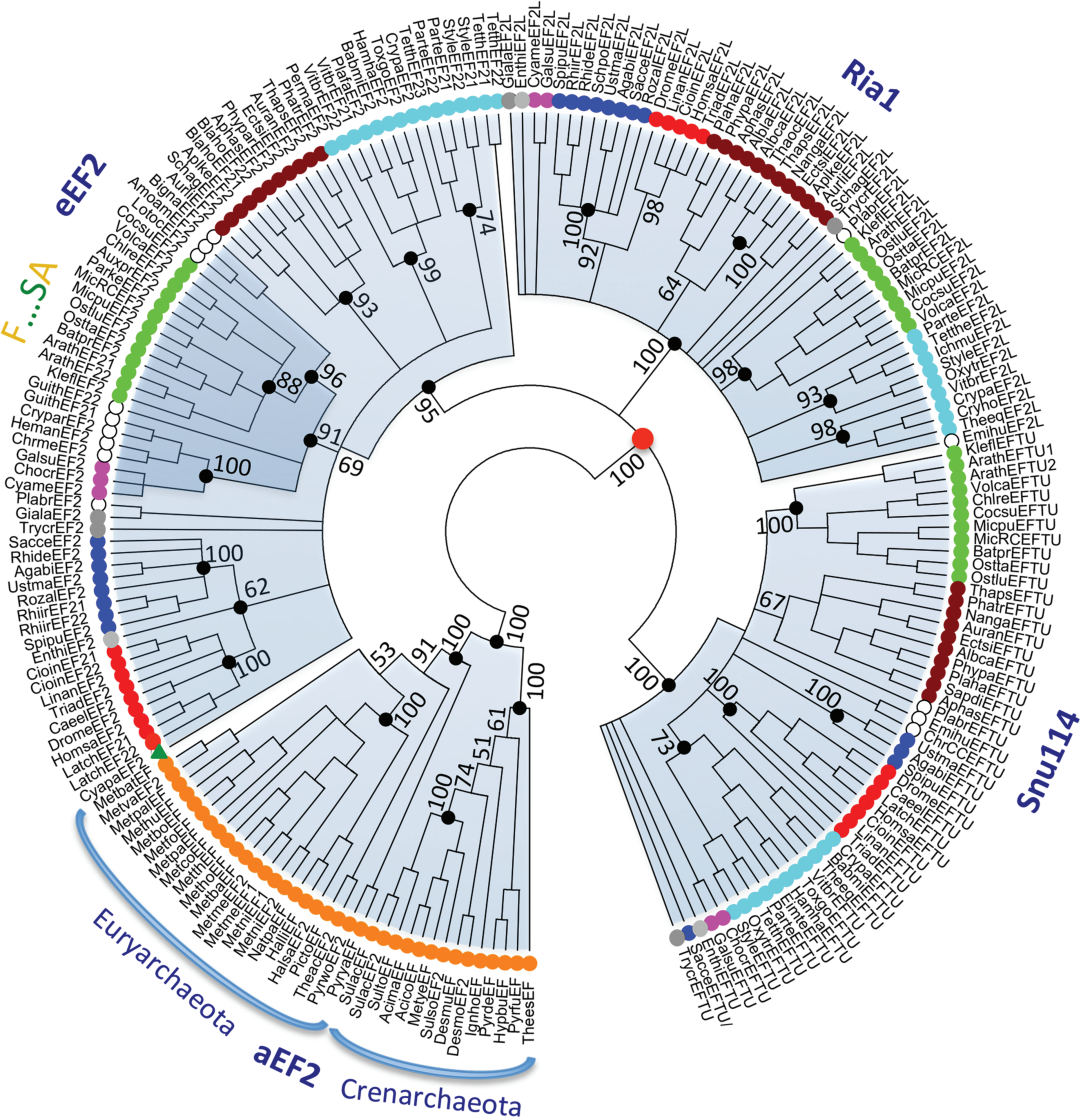
Evolutionary relationship between clades of EF2. A bootstrap consensus tree was generated by conducting a maximum likelihood analysis using RAxML and 1000 bootstrap rounds. Branches corresponding to partitions reproduced in fewer than 50% of the 1000 bootstrap replicates were collapsed. A separate Bayesian inference analysis was carried out using the program MrBayes, which resulted in a tree similar to that shown in Fig. 7. Black dots at nodes in the RAxML consensus tree indicate maximum statistical support (*P* = 1) in the Bayesian inference analysis. The Bayesian interference analysis was run for 5 000 000 generations and the average standard deviation of split frequencies between the resulting trees was 0.010103.

**Figure 9:**
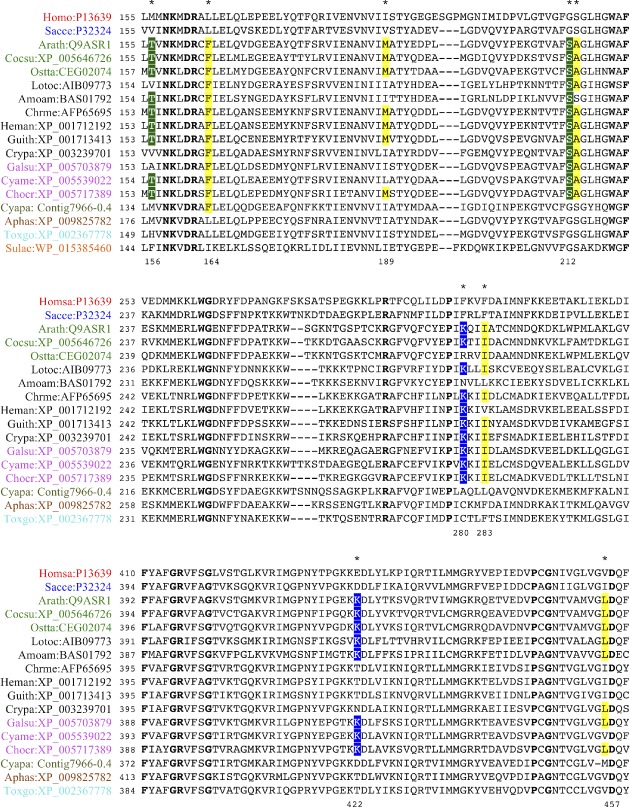
Sequence signatures in EF2 are shared not only between Chloroplastida and Rhodophyceae but also with Crytophyta and Rhizaria. Conserved residues in EF2 are marked by bold type. Asterisks mark synapomorphies identified by Stiller et al. (2001). Homo, *Homo sapiens* (Metazoa); Sacce, *Saccharomyces cerevisiae* (fungi); Arath, *Arabidopsis thaliana* (Streptophyta, Chloroplastida); Cocsu, *Coccomyxa subellipsoidea* C‐169 (Chlorophyta, Chloroplastida); Ostta, *Ostreococcus taurus* (Chlorophyta, Chloroplastida); Lotoc, *Lotharella oceanica* (Rhizaria); Amam, *Amorphochlora amoebiformis* (Rhizaria); Chrme, *Chroomonas mesostigmatica* CCMP1168 (Cryptophyta); Heman, *Hemiselmis andersenii* (Cryptophyta); Guith, *Guillardia theta* (Cryptophyta); Crypa, *Cryptomonas paramecium* (Cryptophyta); Cyapa, *Cyanophora paradoxa* (Glaucophyta); Aphas, *Aphanomyces astaci* (Oomycetes, Stramenopiles); Toxgo, *Toxoplasma gondii* (Apicomplexa, Alveolata); and Sulac, *Sulfolobus acidocaldarius* (Archaea).

To evaluate the evolutionary link between Chloroplastida and Rhodophyceae in more detail, we repeated the analysis of eEF2 proteins (Moreira et al. 2000), but with more sequences and from additional supergroups. Although our analysis confirmed that Chloroplastida are related to Rhodophyceae, it showed that the relationship is paraphyletic (Fig. 7; statistics for major branches following maximum likelihood and Bayesian inference analyses, respectively, are shown in Fig. 8). Thus, within the eEF2 clade, Cryptophyta and Rhizaria were also present, and only the latter group appeared as a direct sister to Chloroplastida. The sequence of the glaucophyte *Cyanophora paradoxa* was placed outside this clade (Fig. 7). Stiller et al. (2001) identified sequence signatures in eEF2 that are common to Chloroplastida and Rhodophyceae. We found these synapomorphies to be present also in eEF2 sequences from Cryptophyta and Rhizaria (Fig. 9), which are supergroups with secondary and tertiary plastids. Intriguingly, the same signature motifs were absent from *C. paradoxa*, which is equipped with a primary plastid (Fig. 9).

## Discussion

### Signs of gene duplications at the time of the last common eukaryotic ancestor

The phylogenetic trees of all P‐type ATPase subfamilies had a complicated architecture, with each eukaryotic supergroup being represented in more than one clade. However, this mirrored property of eukaryotic clades provides evidence that both P‐type ATPase genes were duplicated very early in eukaryotic evolution, before the divergence of the present eukaryotic supergroups (Figs 1, 2, 4 and 5). A similar duplication event has recently been observed in P4 ATPase flippases, a third P‐type ATPase subfamily (Palmgren et al. 2019 *)*.

As the ancient duplication events in P2A, P5A and P4 ATPases appear to have happened only once, it raises the question of whether the P‐type ATPase genes were duplicated together. Three possible scenarios explain how this could have happened. One possibility would be a local duplication, with the genes that were duplicated being present in the same chromosome region. However, this seems unlikely, as diverse P‐type ATPase genes in prokaryotic genomes do not tend to reside in close proximity to each other and would not explain the duplication of genes unrelated to P‐type ATPases. The second possibility would be that several independent duplications occurred in succession. While this remains a possibility, it does not clarify why the duplications occurred in multiple genes and only once (or twice) in each gene. The third possibility would be that a whole genome duplication through polyploidization increased the number of all genes in the genome simultaneously. An early whole genome duplication would have been expected to leave signs on multiple other genes and result in the same scenario as observed for P‐type ATPases. Indeed, the early evolution of eukaryotic EF2 involved a gene duplication event that predated the last common ancestor of eukaryotes (Atkinson 2015). Other gene families unrelated to P‐type ATPases, such as the families of Hsp70 (Boorstein et al. 1994, Gupta et al. 1994), Hsp90 (Gupta 1995), α‐ and β‐tubulin (Nozaki et al. 2003), RNA polymerases (Zong et al. 2009), argonaute proteins (Swarts et al. 2014) and phosphatidylinositol‐3‐kinases (Philippon et al. 2015), also appear to have diverged in the evolutionary history of eukaryotes as a result of a single ancient gene duplication event. The chaperonin CCT (chaperonin containing TCP‐1) family also expanded before the appearance of the major eukaryotic supergroups, but with additional duplications occurring (Archibald et al. 2001). Comparative genomic analysis of genes, which form clusters of paralogs in all or most eukaryotic lineages but not in prokaryotes, supports the notion that an extensive paralogization involving thousands of genes occurred early in eukaryotic evolution (Makarova et al. 2005). A careful phylogenetic analysis of many other eukaryotic gene families is required to support the notion that a polyploidization event occurred at the time of the last common eukaryotic ancestor with subsequent differential loss of paralogs in descendant lineages.

### The use of synapomorphies in evolutionary studies

Phylogenetic studies that rely on single gene analyses and on concatenation of multiple single genes have proven successful for determining evolutionary relationships among closely related organisms, but have failed to resolve the deep root of eukaryotic evolution. In this study, the full complement of isoforms of a small subset of well‐characterized genes was included in a detailed analysis, which involved identifying sequence synapomorphies, and resulted in phylogenetic trees with a higher resolution of the eukaryotic root. The synapomorphies identified are single amino acid substitutions, single amino acid indels, and differences in 3–4 amino acid motifs. Are the sequence signatures identified here less convincing because they are short? Several lines of evidence point to the evolutionary significance of the single amino acid synapomorphies identified. First, the sequence signatures uniting Chlorophyceae with supergroups of SAR to the exclusion of Rhodophyceae are present in all sequences analyzed. Similarly, the synapomorphies that are indicative of early gene duplications are conserved in all sequences analyzed with extremely few exceptions (e.g. in Alveolata), as indicated in Figs 4 and 5 and Tables S1 and S2. Such conservation indicates that they are not randomly preserved but have functional significance. In support of this notion, the motifs are situated in conserved parts of the basic catalytic machinery of the pumps. Second, the synapomorphies are present in regions of the pumps that are part of either the basic pumping machinery or relate to charged residues in transmembrane segments. A conserved charged residue in the transmembrane region of an ion pump is likely to impact the pump's ability to bind and/or release the transported ligand (Morth et al. 2011, Palmgren and Nissen 2011).

### On the origin of plants

**Figure 10:**
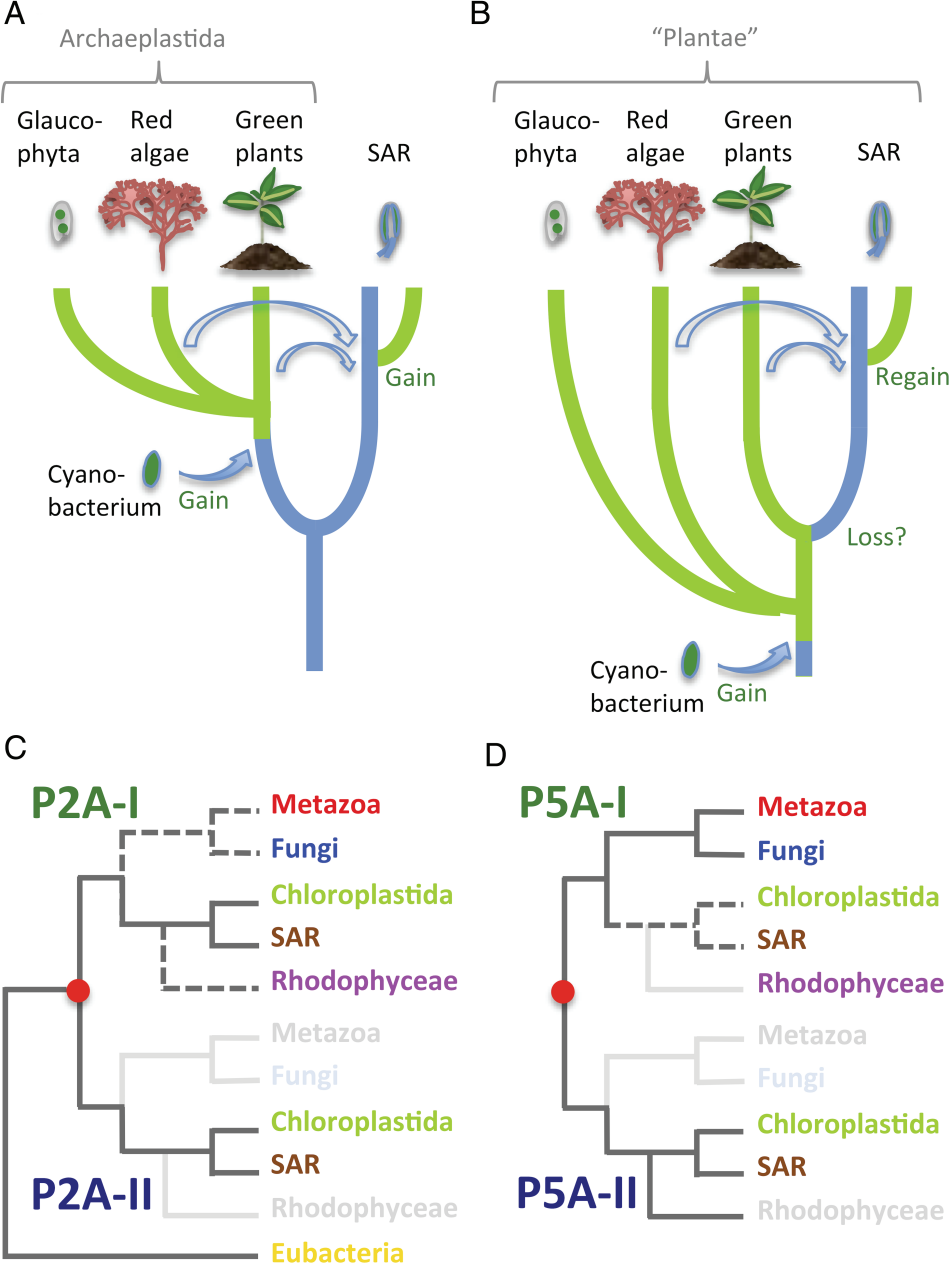
Models for evolution of eukaryotic supergroups. (A and B) Models for evolution of photosynthetic eukaryotes. (A) Monophyletic versus (B) paraphyletic relationship of red algae (Rhodophyceae) and green plants (Chloroplastida). Green branches are lineages with photosynthesis. Blue branches are lineages without photosynthesis. Filled arrows indicate gain of photosynthesis following primary endosymbiosis with a cyanobacterium. Open arrows show gain (A) or regain (B) of photosynthesis following secondary endosymbiosis with red or green algae. The SAR megagroup comprises the Stramenopiles, Alveolata, Rhizaria, Cryptophyta and Haptophyta supergroups. Some lineages within these supergroups carry out photosynthesis, whereas others do not. Secondary transfer of red and green plastids into the SAR could have involved serial events of endosymbiosis (Stiller 2014). Euglenoids (not shown in the figure) also have secondary green plastids but are not part of the SAR complex (Ebenezer et al. 2019). (C and D) **M**odels for the evolution of P2A (C) and P5A (D) ATPases. Phylogenetic analyses in combination with the identification of synapomorphies suggest that a gene duplication event occurred at the time of the last eukaryotic common ancestor (LECA). After duplication, pumps were lost in some lineages (marked by light gray text) and maintained in others (colored text). Dashed lines represent connections that did not receive significant statistical support in the phylogenetic analysis. The red dot indicates an early gene duplication event.

What is a plant? A common definition is that it is an eukaryotic organism with a primary plastid (Adl et al. 2019). Primary plastids are surrounded by two membranes that originated from a photosynthesizing cyanobacterium living in an endosymbiotic relationship within a eukaryotic cell (Ponce‐Toledo et al. 2017, Sánchez‐Baracaldo et al. 2017). According to this definition, Chloroplastida (green plants, including land plants (Streptophyta) and green algae (Chlorophyta)), glaucophytes (Glaucophyta), and red algae (Rhodophyceae), are plants and united in ‘Archaeplastida’ (Adl et al. 2019; Fig. 10A). Many other eukaryotes carry out photosynthesis, but these have plastids with three or more membranes that were derived from eukaryotes with primary plastids, such as red or green algae, that were engulfed by other eukaryotic cells (Delwiche 1999, Keeling 2013). Such organisms with three or more plastid membranes are found in Stramenopiles (including diverse groups, such as photosynthetic brown algae, diatoms and non‐photosynthetic oomycetes), Alveolata (including photosynthetic dinoflagellate algae and non‐photosynthetic ciliates), and the groups of mostly unicellular Rhizaria, Cryptophyta and Haptophyta. Together, Stramenopiles, Alveolata, Rhizaria, Cryptophyta and Haptophyta, form the eukaryotic megagroup SAR (Burki et al. 2008; Fig. 10A) and are not considered to be plants.

Independent support for the monophyly of Archaeplastida, and the exclusion of other eukaryotic supergroups from this clade, came from sequence analysis of the nuclear‐encoded protein eukaryotic (eEF2; Moreira et al. 2000) and was seemingly confirmed by analysis of 143 orthologous nuclear proteins (30 113 amino acid positions) from 39 species (Rodríguez‐Ezpeleta et al. 2005). However, in neither of these studies were Cryptophyta, Haptophyta and related taxa included. Several studies have subsequently reported phylogenetic trees based on multiple nuclear genes from a broad collection of species, but notably they vary considerably with respect to the position of Chloroplastida, which does not consistently group with red algae, but often with Cryptophyta, Haptophyta and Rhizaria (reviewed in Mackiewicz and Gagat 2014). As an alternative model to the monophyly of red algae and green plants, Nozaki et al. (2003) proposed that red algae and green plants have a paraphyletic origin, and that red algae are no more related to green plants than are non‐photosynthesizing members of SAR (as depicted in Fig. 10B). According to this model, photosynthesis was lost in the branch that led to SAR but was regained independently in some but not all lineages by secondary endosymbiosis (Fig. 10B). An overall interpretation of P2A and P5A ATPase phylogenies and synapomorphies (presented in Figs 10C,D) support this model.

Based on a phylogenetic analysis of eEF2 sequences (Moreira et al. 2000), it was concluded that Chloroplastida and Rhodophyceae have a common monophyletic origin to the exclusion of other supergroups. However, Stiller et al. (2001) analyzed synapomorphies in eEF2 and concluded that an apparent signal uniting Chloroplastida and Rhodophyceae was only present in one contiguous section of EF2, and that the gene required more thorough investigation before it could be considered to carry evidence for a red/green monophyly. The findings of our study give little support to the early contention by Moreira et al. (2000). Although we could confirm that Chloroplastida and Rhodophyceae are evolutionarily related to each other, this relationship was not to the exclusion of SAR supergroups. In this study, by contrast, the phylogenetic analysis of two P‐type ATPase subfamilies, which are all encoded by non‐photosynthetic nuclear genes, revealed strong links between Chloroplastida and SAR, but in no cases with Rhodophyceae. Most notably, and in strong support of a close relationship between Chloroplastida and SAR to the exclusion of Rhodophyceae, we identified two P‐type ATPase synapomorphies that unite Chloroplastida with Stramenopiles, Cryptophyta, Haptophyta and Rhizaria, but are not found in Rhodophyceae. The first is a KQF motif in TM1 of the clade of P2A‐I ATPases, the second and the third are a RMxE motif in the A domain and a GDG(A/G) motif in the P domain, respectively, of the clade of P5A‐II ATPases. This does not rule out the possibility that Rhodophyceae and Chloroplastida share a common origin but suggests that Rhodophyceae is an early lineage that branched off the evolutionary tree before the emergence of Chloroplastida, Stramenopiles, Cryptophyta, Haptophyta and Rhizaria (Fig. 10B). An early origin of Rhodophyceae is supported by the fossil record: fossils of multicellular rhodophytes have been identified in 1.6 billion years old sedimentary rocks, a presence that predates that of any other known eukaryotic organism (Bengtson et al. 2017).

There is now consensus that plastids evolved from a single cyanobacterial ancestor (Ponce‐Toledo et al. 2017, Sánchez‐Baracaldo et al. 2017). However, it has been noted on several occasions that a close evolutionary relationship between Chloroplastida and Rhodophyceae is questionable, as Chloroplastida does not consistently group with Rhodophyceae in phylogenetic studies, but often with Cryptophyta, Haptophyta and Rhizaria (reviewed in Mackiewicz and Gagat 2014). Furthermore, in large‐scale studies, signs of monophyly between Chloroplastida and Rhodophyceae vanish when genes of cyanobacterial origin are excluded from the phylogenetic analysis (Katz and Grant 2015). This suggests that Chloroplastida and Rhodophyceae have primary plastids in common but otherwise are distantly related. Primary plastids could have been acquired at a very early stage in evolution and subsequently lost in some lineages, and photosynthesis could, in later separate events, have been regained following secondary endosymbiosis. The fact that Chloroplastida and Rhodophyceae both have primary plastids therefore does not exclude the possibility that Chloroplastida are more closely related to SAR than to Rhodophyceae (Fig. 10B).

Although loss of photosynthesis and concomitant plastid reduction is common, even in land plants, there are so far no reports of complete plastid loss from an organism whose ancestors bore primary plastids (Stiller 2014). In this study, one class of Stramenopiles appeared more often closely linked to Viridiplantae sequences than other Stramenopiles groups, namely the three Labyrinthulomycete representatives of the order Thraustochytriida: *Auranthiochytrium limacinum*, *Schizochytrium aggregatum* and *Aplanochytrium kerguelense*. Their sister relationship with Viridiplantae was in particular mirrored in the P2A ATPase tree. Labyrinthulomycetes are single cell parasites and heterotrophic decomposers that are related to the stramenopilean class Oomycetes, which are fungus‐like heterotrophs. Labyrinthulomycetes were previously considered as belonging to Amoebozoa (Sullivan et al. 2013), do not have chloroplasts and appear not to have anything in common with plants. However, one characteristic suggests that the ancestor of this group of organisms was photosynthetic. Zoospores in the genus Labyrinthula of the order Labyrinthulida have eyespots (Perkins and Amon 1969), that resemble similar structures in other Stramenopiles and in alveolate dinoflagellates having characteristics of being reduced chloroplasts (Dodge 1984, Motomura 1994). Thus, although eyespots are absent in the orders Thraustochytriida and Aplanochytriida of Labyrinthulomycote included in this study (Chamberlain and Moss 1988, Porter 1990), it cannot be ruled out that a common ancestor of Viridiplantae and Labyrinthulomycetes had chloroplasts, and that these subsequently were lost in the ancestor of Labyrinthulomycetes (Tsui et al. 2009, Derelle et al. 2016). Indeed, it has been suggested that the ancestors of all Stramenopiles (and Alveolata) had primary plastids that were lost (Nozaki et al. 2003).

The proteomes of *Thalassiosira* and *Phaeodactylum* (Stramenopila), both of which are diatoms with plastids of red algal origin, contain more than a thousand genes that appear to be more closely related to green algae than to red algae (Moustafa et al. 2009). These data were explained by a green algal‐like endosymbiont in the ancestor of Stramenopiles that was subsequently lost and, in some lineages, replaced by a red algal‐like plastid (Moustafa et al. 2009). The presence of genes in diatoms with high similarity to green algae has also been suggested, at least in part, to be the result of phylogenetic artifacts (Woehle et al. 2011, Burki et al. 2012, Stiller 2014). An alternative possibility remains, namely that the green algal‐like sequences were not derived from endosymbiotic or horizontal gene transfer but rather reflect a true sister‐group relationship between green algae and Stramenopiles (Fig. 10B).

## Conclusion and future perspectives

In this work, we present evidence that green plants and red algae, both of which contain primary plastids, are more distantly related to each other than are green plants to eukaryotic supergroups, in which secondary or tertiary plastids are common. To reach this conclusion, we used synapomorphies as a tool to interpret complex phylogenetic trees and resolve controversial branches. This approach should be useful for addressing the hypothesis further in other protein families. Identifying synapomorphies requires detailed knowledge of structure–function relationships in each family. In this regard, we had the benefit of our extensive previous biochemical and structural experience with P‐type ATPases. However, acquiring such knowledge is a major challenge in very large datasets, and therefore currently is the main limitation of such an approach.

## Author contributions

M.P. conceived and designed the study. M.P., D.M.S. and B.M.H. did the phylogenetic analyses. M.P., T.S. and K.B. interpreted the data. M.P. and K.B. wrote the manuscript. All authors approved the manuscript before submission.

## Supporting information


**Fig S1.** Transmembrane segments (TMs) 4, 5, 6, and 8 of SERCA‐like pumps.
**Fig S2.** Residues in transmembrane segment 1 (TM1) that characterize P5A ATPases.
**Table S1.** P2A SERCA‐like proteins in selected organisms.
**Table S2.** P5A ATPase‐like proteins in selected organisms.
**Table S3.** EF2‐like proteins in selected organisms.

## Data Availability

The data of this study were derived from resources available in the public domain: NCBI protein database (https://www.ncbi.nlm.nih.gov/protein); the Joint Genome Institute (JGI) Genome Portal (http://genome.jgi.doe.gov/); the PlantGDB database (http://www.plantgdb.org/PpGDB/cgi-bin/blastGDB.pl#PPpep:Pp1s6_11V6.1); the *Porphyridium purpureum* Genome Project server (http://cyanophora.rutgers.edu/porphyridium/); the Phytozome Plant Genomics Resource (https://phytozome.jgi.doe.gov/pz/portal.html#!search?show=BLAST); the Conifer Genome Network (CGN) Dendrome Database (http://dendrome.ucdavis.edu/resources/blast/); the Mnemiopsis Genome Project Portal (http://dendrome.ucdavis.edu/resources/blast/); the Cyanophora Genome Project server (http://cyanophora.rutgers.edu/cyanophora/blast.php); and the Plantmorphogenesis server (http://www.plantmorphogenesis.bio.titech.ac.jp/~algae_genome_project/klebsormidium/klebsormidium_blast.html).
