# Supplementary material for: Evolution of P2A and P5A ATPases: ancient gene duplications and the red algal connection to green plants revisited
Source: Physiol Plant. 2019 Aug 8;168(3):630–47. doi: 10.1111/ppl.13008 (PMC7065118; doi:10.1111/ppl.13008)
Supplement: Supplementary file 1 — Fig S1. Transmembrane segments (TMs) 4, 5, 6, and 8 of SERCA‐like pumps. Fig S2. Residues in transmembrane segment 1 (TM1) that characterize P5A ATPases. Table S1. P2A SERCA‐like proteins in selected organisms. Table S2. P5A ATPase‐like proteins in selected organisms. Table S3. EF2‐like proteins in selected organisms. [file PPL-168-630-s001.pdf]

Supporting information

Evolution of P2A and P5A ATPases: Ancient gene duplications and the red algal connection to green plants revisited

Michael Palmgren[1,2,*], Danny Mollerup Sørensen[1], Björn M. Hallström[3], Torbjörn Säll[4], and Karin Broberg[2]

[1]Department of Plant and Environmental Sciences, University of Copenhagen, Denmark

[2] Institute of Environmental Medicine, Karolinska Institutet, Stockholm, Sweden

[3]Science for Life Laboratory, KTH - Royal Institute of Technology, Stockholm, Sweden

[4]Department of Biology, Lund University, Lund, Sweden

***Corresponding author**: E-mail: palmgren@plen.ku.dk

Figure S1

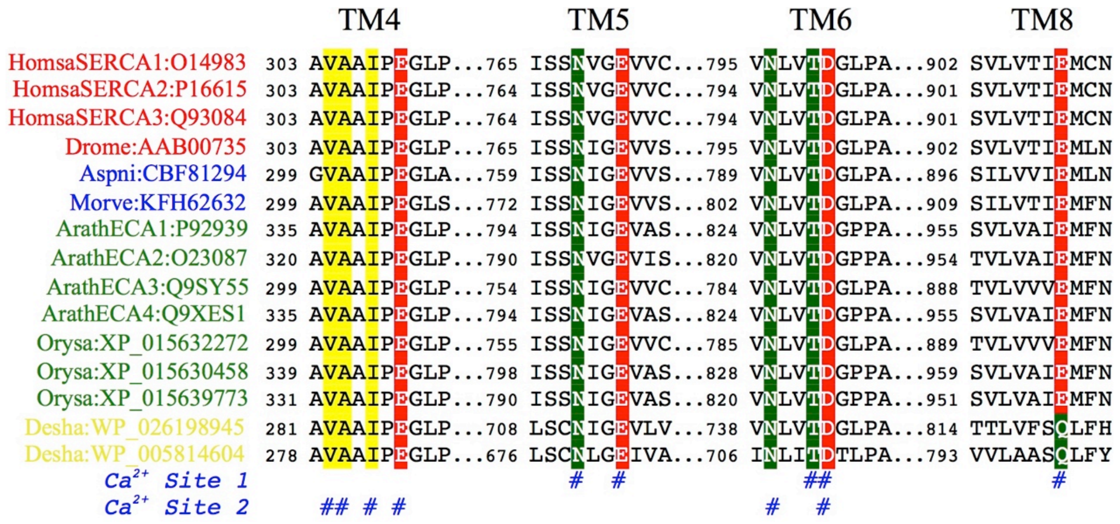

Figure S1. Transmembrane segments (TMs) 4, 5, 6, and 8 of SERCA-like pumps. Residues that contribute to coordinating Ca^{2+} ions in Site 1 and Site 2, respectively, are marked with hash symbols and highlighted. Sequences are from selected organisms (abbreviated names are in parentheses precede accession numbers in the figure): animals (red text), *Homo sapiens* (Homsa) and *Drosophila melanogaster* (Drome); fungi (blue text), *Aspergillus niger* (Aspni) and *Mortierella verticillata* (Morve); land plants (green text), *Arabidopsis thaliana* (Arath) and *Oryza sativa* (Orysa); and bacteria (yellow text), *Desulfitobacterium hafniense* (Desha).

Figure S2

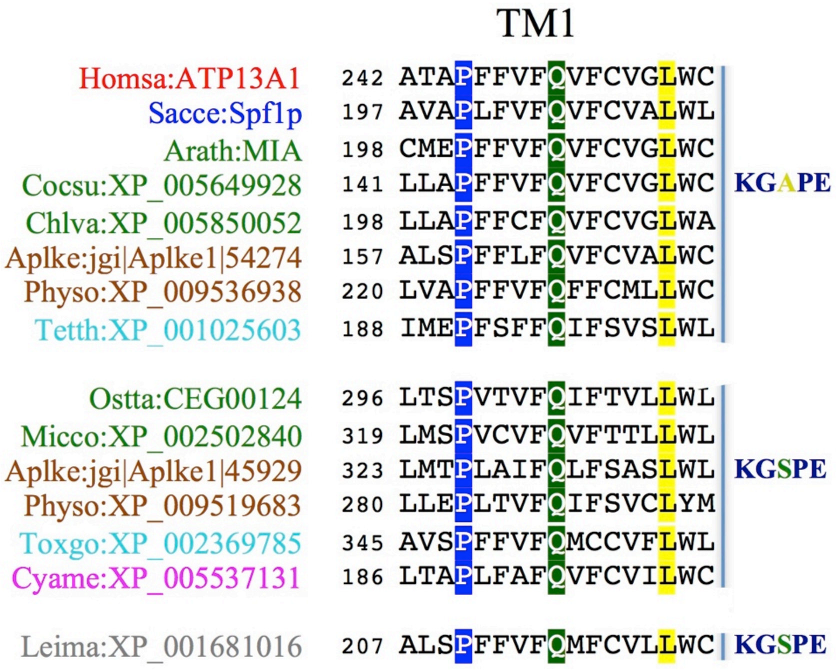

Supplementary Figure 2. Residues in transmembrane segment 1 (TM1) that characterize P5A ATPases. Sequences are from selected organisms (abbreviated names and accession numbers are in parentheses): animals (red text), *Homo sapiens* (Homsa:ATP13A1, Q9HD20); fungi (blue text), *Saccharomyces cerevisiae* (Sacce:Spf1p, Q9HD20); Chloroplastida (green text), *Arabidopsis thaliana* (Arath:MIA, Q9LT02), *Coccomyxa subellipsoidea* (Cocsu, XP_005649928), *Chlorella variabilis* (Chlva, XP_005850052), *Ostreococcus tauri* (Ostta, CEG00124), and *Micromonas commode* (Micco, XP_002502840); Stramenopiles (brown text), *Aplanochytrium kerguelense* (Ak, jgi|Aplke1|54274), and *Phytophthora sojae* (Physo, XP_009519683); Alveolata (turquoise text), *Toxoplasma gondii* (Toxgo, XP_002369785) and *Tetrahymena thermophila* (Tetth, XP_001025603); Rhodophyceae (cyan text), *Cyanidioschyzon merolae* (Cyame, XP_005537131); and Discobids (gray text), *Leishmania major* (Leima, XP_001681016).

Table S1

P2A SERCA-like proteins in selected organisms

Taxonomy	Species	Acc. Nr.	Abbrev. Name	P domain	TM6	TM1	Comments
				Synapomorphies			
**Eukaryota**							
**Opisthokonta**							
**Metazoa**							
**Deuterostomia**							
**Chordata**							
**Mammalia**							
	Homo sapiens	O14983	HomsaSRCA1	GIAMG-SG	TDGLPA	EQFED	
		P16615	HomsaSRCA2	GIAMG-SG	TDGLPA	EQFED	
		Q93084	HomsaSRCA3	GIAMG-SG	TDGLPA	EQFED	
**Aves (birds)**							
	Gallus gallus	P13585	GalgaSRCA1	GIAMG-SG	TDGLPA	EQFED	
		Q03669.2	GalgaSRCA2	GIAMG-SG	TDGLPA	EQFED	
		Q9YGL9	GalgaSRCA3	GIAMG-SG	TDGLPA	EQFED	
**Reptilia**							
	Chelonia mydas	XP_007072304.1	ChemySRCA1	GIAMG-SG	TDGLPA	EQFED	
		XP_007058345.1	ChemySRCA2	GIAMG-SG	TDGLPA	EQFED	
		XP_007054215.1	-				Lacking N-terminal sequence including TM1 and T2
**Amphibia**							
	Xenopus tropicalis	XP_012810385.1	XentrSRCA1	GIAMG-SG	TDGLPA	EQFED	
		XP_004910568.1	XentrSRCA2	GIAMG-SG	TDGLPA	EQFED	
		NP_001072333.1	XentrSRCA3	GIAMG-SG	TDGLPA	EQFED	
**Actinopterygii (ray-finned fish)**							
	Danio rerio	NP_001007030.1	DanreSCA1a	GIAMG-SG	TDGLPA	EQFED	
		NP_001071001.1	DanreSCA1b	GIAMG-SG	TDGLPA	EQFED	
		XP_021333895.1	DanreSRCA2	GIAMG-SG	TDGLPA	EQFED	
		XP_697108.5	DanreSRCA3	GIAMG-SG	TDGLPA	EQFED	
**Tunicata**							
	Ciona intestinalis	XP_002127180.1	CioinSRCA1	GIAMG-SG	TDGLPA	EQFED	
		XP_009861043.1	CioinSRCA2	GIAMG-SG	TDGLPA	EQFED	
	Molgula tectiformis	BAG71430.1	MolteSRCA1	GIAMG-SG	TDGLPA	EQFED	
		BAG71432.1	-				
**Protostomia**							
**Mollusca**							
**Bivalvia**							
	Crassostrea gigas	XP_011425752.1	CragiSRCA	GIAMG-SG	TDGLPA	EQFDD	
**Gastropoda**							
	Lottia gigantea	XP_009051621.1	LotgiSRCA	GIAMG-SG	TDGLPA	EQFDD	
**Brachiopoda**							
**Lingulata**							
	Lingula anatina	XP_013421498.1	LinanSRCA	GIAMG-SG	TDGLPA	EQFDD	
**Nematoda**							
**Chromadorea**							
	Caenorhabditis elegans	G5EEK8	CaelSRCA	GISMG-SG	TDGLPA	EQFDD	
**Arthropoda**							
**Branchiopoda**							
	Daphnia pulex	EFX65740.1	DappuSRCA	GIAMG-SG	TDGLPA	EQFDD	
**Insecta**							
	Drosophila melanogaster	AAB00735.1	DromeSRCA	GIAMG-SG	TDGLPA	EQFDD	
**Arachnida**							
	Stegodyphus mimosarum	KFM58607.1	StemiSRCA	GIAMG-SG	TDGLPA	EQFDD	
**Cnidaria**							
**Anthozoa**							
	Nematostella vectensis	XP_001639528.1	NemveSRCA	GVAMG-SG	TDGPPA	EQFDD	
**Placozoa**							
	Trichoplax adhaerens	XP_002115035.1	TriadSRCA	GVAMG-SG	TDGLPA	EQFDD	
**Choanoflagellida**							
	Salpingoeca rosetta	XP_004992033.1	SalroSRCA	GVAMG-SG	TDGLPA	EQFDD	
**Ichthyosporea**							
	Capsaspora owczarzaki ATCC 30864	XP_004344407.2	CapowSRCA	GVAMG-SG	TDGLPA	EQFDD	
**Fungi**							
**Ascomycota**							
**Eurotiomycetes**							
	Aspergillus nidulans FGSC A4	CBF81294.1	AspniSRCA	GVAMG-TG	TDGLPA	EQFKD	
	Neosartorya fischeri NRRL 181	XP_001257791.1	NeofiSRCA	GVAMG-TG	TDGLPA	EQFKD	
	Byssochlamys spectabilis No. 5	GAD96377.1	ByssPSRCA	GVAMG-SG	TDGLPA	EQFKD	
	Coccidioides immitis RS	XP_001243422.2	CocimSRCA	GVAMG-SG	TDGLPA	EQFKD	
**Leotiomycetes**							
	Pseudogymnoascus sp. VKM F-4514 (FW-929)	KFY41655.1	PsepaSRCA	GVAMG-SG	TDGLPA	EQFKD	
**Sordariomycetes**							
	Fusarium oxysporum Fo47	EWZ50082.1	FusoxSRCA	GVAMG-SG	TDGLPA	EQFKD	
	Metarhizium robertsii ARSEF 23	XP_007823469.2	MetroSRCA	GVAMG-SG	TDGLPA	EQFKD	
**Basidiomycota**							
**Agaricomycetes**							
	Agaricus bisporus var. burnettii JB137-S8	XP_007326281.1	AgabiSRCA	GVAMG-SG	TDGLPA	EQFKD	
	Coprinopsis cinerea okayama7#130	XP_001833748.2	CopciSRCA	GVAMG-SG	TDSLPA	EQFKD	
	Schizophyllum commune H4-8	XP_003035126.1	SchcoSRCA	GVAMG-SG	TDSLPA	EQFKD	
**Tremellomycetes**							
	Tremella mesenterica DSM 1558	XP_007005106.1	TremeSRCA	GIAMG-SG	TDGLPA	AQFKD	
	Trichosporon oleaginosus	KLT45999.1	TriolSRCA	GIAMG-SG	TDGGPA	AQFED	
	Trichosporon asahii var. asahii CBS 8904	EKD03529.1	TriasSRCA	GIAMG-SG	TDAAPA	AQFED	
**Ustilaginomycetes**							
	Ustilago maydis 521	XP_011388455.1	UstmaSRCA	GIAMG-SG	TDGLPA	DQFKD	
	Pseudozyma antarctica T-34	GAC72184.1	PseanSRCA	GIAMG-SG	TDGLPA	EQFKD	
**Glomeromycota**							
**Glomeromycetes**							
	Rhizophagus irregularis	ESA01669.1	RhiirSRCA	GIAMG-DG	TDGLPA	EQFKD	
**Zygomycota**							
**Mucoromycotina**							
	Mortierella verticillata NRRL 6337	KFH62632.1	MorveSRCA	GIAMG-SG	TDGLPA	EQFKD	
	Mucor circinelloides f. circinelloides 1006PhL	EPB84536.1	MucciSRCA1	GIAMG-SG	TDGLPA	EQFQD	
		EPB85748.1	MucciSRCA2	GIAMG-SG	TDGLPA	DQFKD	
	Rhizopus microsporus	CEJ01628.1	RhimiSRCA	GVAMG-SG	TDGLPA	EQFKD	
**Chytridiomycota**							
**Chytridiomycetes**							
	Spizellomyces punctatus DAOM BR117	KNC99029.1	SpipuSRCA	GIAMG-TG	TDGLPA	EQFKD	
**Blastocladiomycota**							
**Blastocladiomycetes**							
	Allomyces macrogynus ATCC 38327	KNE68426.1	AllmaSRCA	GVAMG-SG	TDGLPA	EQFQD	
**Cryptomycota**							
	Rozella allomycis CSF55	EPZ34186.1	RozalSRCA	GVSMG-SG	TDGLPA	EQFED	
**Viridiplantae**							
**Streptophyta**							
**Embryophyta**							
**Magnoliophyta**							
**Eudicotyledons**							
	Arabidopsis thaliana	Q9SY55	ArathSRCA; AtECA3	GIAMG-SG	TDGLPA	KQFDD	
		P92939	ArathECA1;AtECA1	GVAMGISG	TDGPPA	EQFND	
		O23087	ArathECA2; AtECA2	GIAMGITG	TDGPPA	EQFDD	
		Q9XES1	ArathECA4; AtECA4	GVAMGISG	TDGPPA	EQFND	
	Solanum tuberosum	XP_006347865.1	SoltuSRCA	GIAMG-SG	TDGLPA	KQFND	
		XP_006363343.1	-				
		XP_006339743.1	-				
	Phaseolus vulgaris	XP_007136772.1	PhavuSRCA	GIAMG-SG	TDGLPA	KQFDD	
		XP_007162693.1	PhaveECA1	GVAMGIAG	TDGPPA	DQFND	
		XP_007135282.1	PhaveECA2	GIAMGITG	TDGPPA	EQFDD	
	Vitis vinifera	XP_010664492.1	VitviSRCA	GIAMG-SG	TDGLPA	KQFDD	
		XP_002284552.1	VitviECA1a	GIAMGITG	TDGPPA	DQFND	
		XP_002277306.1	VitviECA1b	GIAMGIAG	TDGPPA	EQFQD	
		XP_002264585.2	VitviECA2	GIAMGITG	TDGPPA	EQFDD	
	Beta vulgaris subsp. vulgaris	XP_010687183.1	BetvuSRCA	GIAMG-SG	TDGLPA	KQFDD	
		XP_010685850.1	BetvuECA1	GIAMGIAG	TDGPPA	DQFND	
		XP_010693884.1	BetvuECA2	GIAMGIAG	TDGPPA	EQFDD	
**Early-diverging eudicotyledons**							
	Nelumbo nucifera	XP_010273141.1	NelnuSRCA	GIAMG-SG	TDGLPA	KQFDD	
		XP_010253395.1	NelnuECA1a	GVAMGVTG	TDGPPA	EQFND	
		XP_010259985.1	NelnuECA1b	GVAMGITG	TDGPPA	EQFND	
		XP_010267484.1	NelnuECA2a	GVAMGITG	TDGPPA	EQFDD	
		XP_010252600.1	NelnuECA2b	GIAMGITG	TDGPPA	EQIDD	
	Aquilegia coerulea	Aquca_060_00052.1	-				Sequence lacks ES in conserved TGES motif
		Aquca_027_00199.1	AqucaECA1a	GIAMGIAG	TDGPPA	EQFND	
		Aquca_010_00647.2	AqucaECA1b	GIAMGIAG	TDGPPA	DQFND	
		Aquca_037_00248.2	AqucaECA1c	GIAMGITG	TDGPPA	EQFDD	
**Monocotyledons**							

	Oryza sativa	XP_015632272.1	-				Sequence is lacking TM3
		XP_015630458.1	OrysaECA1a	GVAMGITG	TDGPPA	EQFDD	
		XP_015639773.1	OrysaECA1b	GVAMGITG	TDGPPA	QQFDD	
	Brachypodium distachyon	XP_003560240.1	BradiSRCA	GIAMG-SG	TDGLPA	KQFDD	
		XP_010228776.1	-				
	Elaeis guineensis	XP_010936144.1	ElaguSRCA	GIAMG-SG	TDGLPA	KQFDD	
		XP_010920750.1	-				
	Musa acuminata subsp. malaccensis	XP_009405782.1	MusacSRCA	GIAMG-SG	TDGLPA	NQFDD	
		XP_009421359.1	-				
**Basal Magnoliophyta**							
	Amborella trichopoda	XP_006857120.1	AmbtrSRCA	GIAMG-SG	TDGLPA	KQFDD	
		XP_006850677.1	AmbtrECA1	GIAMGITG	TDGPPA	DQFND	
		ERN13344.1	AmbtrECA2	GIAMGITG	TDGPPA	EQFDD	
**Lycopodiopsida (club mosses)**							
	Selaginella moellendorffii	XP_002985331.1	SelmoSRCA	GIAMG-SG	TDGLPA	KQFDD	
		XP_002974690.1	SelmoECA1a	GIAMGITG	TDGPPA	EQFDD	
		XP_002968844.1	SelmoECA1b	GIAMGITG	TDGPPA	EQFDD	
		XP_002980453.1	SelmoECA2	GIAMGLSG	TDGAPA	EQFDD	
**Bryophyta (mosses)**							
	Physcomitrella patens	XP_001778460.1	PhypaSRCA	GIAMG-SG	TDGLPA	KQFDD	
		XP_001765945.1	PhypaECA1a	GVAMGISG	TDGPPA	EQFDD	
		XP_001778955.1	PhypaECA1b	GVAMGIAG	TDGPPA	EQFDD	
		XP_001765694.1	PhypaECA1c	GVAMGIAG	TDGPPA	EQFDD	
		XP_001759652.1	PhypaECA2	GIAMGITG	TDGAPA	EQFED	
**Klebsormidiophyceae**							
	Klebsormidium flaccidum	kfl00413_0060	KleflSRCA	GIAMG-SG	TDGLPA	KQFDD	
		kfl00112_0080	KleflECA1	GIAMGIAG	TDGPPA	EQFED	
**Chlorophyta**							
**Chlorophyceae**							
	Chlamydomonas reinhardtii	XP_001699486.1	-				Sequence lacks conserved PEGLP and DKTGTL motifs
		XP_001700727.1	ChlreECA1	GVAMGIAG	TDGPPA	EQFED	
	Volvox carteri f. nagariensis	XP_002953236.1	VolcaSRCA	GVAMG-SG	TDGLPA	KQFDD	
		XP_002958852.1	VolcaECA1	GVAMGIAG	TDGPPA	EQFED	
	Coccomyxa subellipsoidea C-169	XP_005646370.1	CocsuSRCA	GIAMG-SG	TDGLPA	KQFDD	
		XP_005652084.1	CocsuECA1	GVAMGIAG	TDGPPA	EQFDD	
	Dunaliella bioculata	P54209.1	DunbiSRCA	GIAMG-SG	TDGLPA	KQFDD	
**Trebouxiophyceae**							
	Chlorella variabilis	XP_005844682.1	-				Partial sequence lacking N-terminal part including conserved TGES and DKTGTL motifs
		XP_005847889.1	ChlvaECA1	GVAMGITG	TDGPPA	EQFDD	
	Auxenochlorella protothecoides	XP_011396861.1	-				Partial sequence lacking N-terminal sequences and conserved DPPR motif
		XP_011397223.1	AuxprECA1	GIAMGVTG	TDGPPA	QQFDD	
**Mamiellophyceae**							
	Micromonas pusilla CCMP1545	XP_003055417.1	MicpuSRCA	GIAMG-SG	TDGLPA	KQFDD	
		XP_003062749.1	MicpuECA1	GIAMGITG	TDGPPA	EQFDD	
	Micromonas commoda	XP_002507319.1	MicRCSRCA	GIAMG-SG	TDGLPA	KQFDD	
		XP_002508654.1	-				
	Ostreococcus tauri	CEF97299.1	OsttaSRCA	GIAMG-SG	TDGLPA	KQFDD	
		CEF96798.1	OsttaECA1	GIAMGIAG	TDGPPA	EQFDD	
	Ostreococcus lucimarinus CCE9901	XP_001417061.1	OstluSRCA	GIAMG-SG	TDGLPA	KQFDD	
		XP_001415608.1	-				
	Bathycoccus prasinos	XP_007512204.1	BatprSRCA	GISMG-SG	TDGLPA	KQFDD	
		XP_007515588.1	BatprECA1	GISMGIAG	TDGPPA	EQFDD	
**Rhodophyta**							
**Florideophyceae**							
	Gracilariopsis chorda	PXF48149.1	GrachSRCA	GIAMG-SG	TDGLPA	EQFED	
	Chondrus crispus	XP_005715614.1	ChocrSRCA	GIAMG-SG	TDGLPA	EQFED	
**Bangiophyceae**							
	Porphyra umbilicalis	OSX81029.1	-				Partial sequence lacking N-terminal part including QF.D motif and withl KGAFE instead of KGAPE
	Galdieria sulphuraria	XP_005707399.1	GalsuSRCA	GIAMG-SG	TDGLPA	EQFQD	Sequence lacking many conserved residues - primarily glycines - that are also lacking in Excavata
		XP_005704597.1	-				Sequence with several deletions including conserved KGAPE motif
	Porphyridium purpureum	evm.model.contig_3542.2	PorpuSRCA	GIAMG-SG	TDGLPA	EQFKD	
		evm.model.contig_2190.8	-				
	Cyanidioschyzon merolae strain 10D	XP_005539258.1	CyameSRCA	GISMG-TG	TDGLPA	KQFQD	
		XP_005537020.1	-				Sequence with several internal deletions
**SAR**							
**Stramenopiles**							
**Oomycetes**							
	Phytophthora parasitica INRA-310	XP_008894556.1	PhyparSRCA	GIAMGITG	TDGPPA	EQFDD	
	Aphanomyces astaci	XP_009821873.1	AphasSRCA	GIAMGISG	TDGPPA	EQFED	
	Saprolegnia diclina VS20	XP_008609540.1	SapdiSRCA	GIAMGISG	TDGPPA	EQFDD	
	Albugo laibachii Nc14	CCA24190.1	AlblaSRCA	GIAMGITG	TDGPPA	EQFDD	
	Albugo candida	CCI41983.1	AlbcaSRCA	GIAMGITG	TDGPPA	EQFDD	
	Plasmopara halstedii	CEG39798.1	PlahaSRCA	GIAMGITG	TDGPPA	EQFDD	
**Bacillariophyta**							
	Fragilariopsis cylindrus CCMP 1102	jgi|Fracy1|210916	FracySRCA1	GIAMGISG	TDGPPA	EQFDD	
		jgi|Fracy1|188142	FracySRCA2	GVAMG-TG	TDGPPA	EQFED	
	Pseudo-nitzschia multiseries CLN-47	jgi|Psemu1|34389	PsemuSRCA1	GIAMGISG	TDGPPA	EQFDD	
		jgi|Psemu1|187628	PsemuSRCA2	GVAMG-TG	TDGPPA	EQFED	Lacking N-terminal sequence
	Thalassiosira pseudonana CCMP1335	XP_002286890.1	ThapsSRCA1	GIAMGLTG	TDGPPA	EQFDD	
		XP_002294029.1	ThapsSRCA2	GVAMGISG	TDGPPA	HQFDD	
		XP_002288284.1	ThapsSRCA3	GVAMGISG	TDGPPA	EQFDD	
	Phaeodactylum tricornutum CCAP 1055/1	XP_002183209.1	PhatrSRCA	GIAMGITG	TDGPPA	EQFED	Lacking N-terminal sequence
**Eustigmatophyceae**							
	Nannochloropsis gaditana CCMP526	XP_005853959.1	NangaSRCA	GVAMGITG	TDGPPA	EQFED	
**Blastocystis**							
	Blastocystis hominis	XP_012895489.1	BlahoSERCA1	GVAMGVSG	TDGPPA	EQFDD	
		XP_012895122.1	BlahoSERCA2	GVAMGVSG	TDGPPA	EQFDD	
		XP_012895050.1	BlahoSERCA3	GVAMGVSG	TDGPPA	EQFED	
**Pelagophycea**							
	Aureococcus anophagefferens	XP_009039668.1	AuranSRCA	GIAMGITG	TDGPPA	SQFED	Lacking N-terminal sequence
		XP_009038985.1	AuranECA1	GIAMGITG	TDGPPA	EQFDD	
**Labyrinthulomycetes**							
	Aplanochytrium kerguelense	jgi|Aplke1|67481	AplkeSRCA	GIAMG-SG	TDGLPA	KQFED	
		jgi|Aplke1|91638	AplkeECA 1	GVAMGITG	TDGPPA	EQFDD	
	Aurantiochytrium limacinum ATCC MYA-1381	jgi|Aurli1|84578	AurliSRCA	GIAMG-SG	TDGLPA	KQFED	
		jgi|Aurli1|43451	AurliECA1	GVAMGIAG	TDGPPA	EQFDD	
	Schizochytrium aggregatum ATCC 28209	jgi|Schag1|80987	SchagSRCA	GIAMG-SG	TDGLPA	?	Sequence is lacking N-terminal part including _QF motif
		jgi|Schag1|83554	SchagECA1	GIAMGITG	TDGPPA	EQFDD	
**Alveolata**							
**Chromerida**							
	Vitrella brassicaformis CCMP3155	CEM18264.1	VitbrSRCA1	GIAMGITG	TDGLPA	DQFED	
		CEM04548.1	VitbrSRCA2	GVAMGVTG	TDGLPA	QQFDD	
**Ciliophora**							
	Paramecium tetraurelia strain d4-2	XP_001453966.1	-				
		XP_001447148.1	ParteSRCA2	GIAMGIAG	TDGLPA	EQFED	
	Tetrahymena thermophila SB210	XP_001030432.2	TetthSRCA	GIAMGISG	TDGLPA	EQFED	
	Stylonychia lemnae	CDW82171.1	StyleSERCA	GIAMGITG	TDGPPA	EQFED	
	Oxytricha trifallax	EJY74000.1	OxytrSERCA	GIAMGITG	TDGPPA	EQFED	
	Ichthyophthirius multifiliis	XP_004040046.1	IchmuSRCA	GIAMGISG	TDGLPA	EQFED	
**Apicomplexa**							
	Eimeria tenella	XP_013228780.1	EimteSRCA	GVAMGIAG	TDGPPA	EQFQD	
	Toxoplasma gondii ME49	XP_002367953.1	ToxgoSRCA	GVAMGIAG	TDGPPA	EQFQD	
	Babesia microti strain RI	XP_012647248.1	BabmiSRCA	GISMGISG	TDGPPA	AQFED	
	Plasmodium falciparum 3D7	BAD73959.1	-				
**Rhizaria**							
**Cercozoa**							
	Plasmodiophora brassicae	CEO97902.1	PlabrSRCA1	GVAMG-SG	TDGLPA	KQFED	
		CEP01171.1	PlabrSRCA2	GIAMG-SG	TDGLPA	KQFQD	
	Bigelowiella natans CCMP2755	jgi|Bigna1|46678	BignaSRCA	GVAMGIAG	TDGPPA	EQFDD	
**Cryptophyta**							
**Pyrenomonadales**							
	Guillardia theta CCMP2712	XP_005821433.1	GuithSRCA	GVAMG-SG	TDGLPA	KQFDD	
		jgi|Guith1|118062	-				Sequence is lacking C-terminal TM domains
**Haptophyta**							
**Isochrysidales**							
	Emiliania huxleyi CCMP1516	XP_005787448.1	EmihuSRCA1	GVAMGIAG	TDGPPA	QQFDD	C-terminal sequence uncertain - lacking DE motif in last TM segment
		XP_005757745.1	EmihuSRCA2	GIAMG-SG	TDGLPA	AQFDD	
		XP_005781031.1	-				Sequence with deletion including GEPT motif
**Prymnesiales**							
	Chrysochromulina sp. CCMP291	KOO23673.1	ChrCCSRCA1	GIAMGISG	TDGPPA	QQFDD	
		KOO33828.1	ChrCCSRCA2	GVAMGIAG	TDGPPA	AQFDD	
**Discobids**							
**Euglenozoa**							
	Leptomonas pyrrhocoris	KPA73157.1	LeppySRCA	GIAMG-SG	TDGLPA	AQFDD	
	Trypanosoma brucei gambiense DAL972	XP_011773626.1	TrybrSRCA	GIAMG-SG	TDGLPA	AQFED	
	Leishmania braziliensis MHOM/BR/75/M2904	XP_001561691.1	LeibrSRCA	GIAMG-SG	TDGLPA	GQFED	

		Leishmania major strain Friedlin	XP_888512.1	LeimaSRCA	GIAMG-SG	TDGLPA	GQFED
**Eubacteria**							
	**Firmicutes**						
		**Clostridia**					
		Desulfitobacterium hafniense	WP_026198945.1	DeshaSRCA	GISMGKTG	TDGLPA	GQFKD
			WP_005814604	-	GVAMGITG	TDTLPA	AQLKD
		Halothermothrix orenii	WP_015923910.1	HalorSRCA	GVAMGITG	TDGFPA	DQFKD
		Symbiobacterium thermophilum	WP_011195466.1	SymthSRCA	GIAMGRTG	TDGLPA	AQFQD
		Clostridium sulfidigenes	WP_035133117.1	ClosuSRCA	GVAMGITG	TDSFPA	AQLND
		**Tissierellia**					
		Clostridium ultunense	WP_005587188.1	CloulSRCA	GVSMGITG	TDSFPA	AQFND
		**Bacilli**					
		Paenibacillus polymyxa	WP_019687953.1	PaepoSRCA	GIAMGITG	TDGLPA	NQFKD
		Alicyclobacillus ferrooxydans	WP_054967681.1	AlifeSRCA	GIAMGQSG	TDGLPA	NQFRD
		Thermoactinomyces daqus	WP_052154068.1	ThedaSRCA	GIAMGVTG	TDGLPA	DQFKD
		Paenibacillus vortex	WP_006209214.1	PaevoSRCA	GIAMGITG	TDGLPA	NQFKD
		Shimazuella kribbensis	WP_037464649.1	ShikrSRCA	GIAMGKTG	TDGLPA	NQFQD
		Bacillus thermoamylovorans	WP_041848609.1	BacthSRCA	GIAMGITG	TDGLPA	SQFKD
		Brevibacillus massiliensis	WP_026074510.1	BremaSRCA	GIAMGITG	TDGLPA	NQFKD
		**Negativicutes**					
		Pelosinus fermentans JBW45	WP_007953053.1	PelfeSRCA	GIAMGTAG	TDGLPA	AQFQD

Table S2

P5A ATPase-like proteins in selected organisms

Taxonomy	Species	Acc. Nr.	Abbrev. Name	A domain	TM4	N domain		P domain	Comments
**Eukaryota**									
**Opisthokonta**									
**Metazoa**									
**Deuterostomia**									
**Chordata**									
**Mammalia**									
	Homo sapiens	Q9HD20	HomsaP5A1/ATP13A1	QGKLLRTILF	PPELPIE	FASALKR	KGAPE	GDGTND	
**Aves (birds)**									
	Gallus gallus	XP_004950884.1	GalgaP5A1	QGKLLRTILF	PPELPIE	FASALKR	KGAPE	GDGTND	
**Reptilia**									
	Chelonia mydas	EMP30881.1	ChemyP5A1	QGKLLRTILF	PPELPIE	FVSALKR	KGAPE	GDGTND	
**Actinopterygii (ray-finned fish)**									
	Danio rerio	NP_001001403.2	DanreP5A1	QGKLLRTILF	PPELPIE	FTSALKR	KGAPE	GDGTND	
**Sarcopterygii (lobe-finned fish)**									
	Latimeria chalumnae	XP_005991859.1	LatchP5A1	QGKLLRTILF	PPELPIE	FASALKR	KGAPE	GDGTND	
**Protostomia**									
**Mollusca**									
**Bivalvia**									
	Crassostrea gigas	XP_011452247.1	CragiP5A1	QGKLLKTILF	PPELPIE	FASALKR	KGAPE	GDGTND	
**Gastropoda**									
	Lottia gigantea	XP_009056493.1	LotgiP5A1	QGKLLKTILF	PPELPIE	FASALKR	KGAPE	GDGTND	
**Annelida**									
**Polychaeta**									
	Capitella teleta	ELU10095.1	CapteP5A1	QGKLLRTILF	PPELPIE	FASALKR	KGAPE	GDGTND	
**Arthropoda**									
**Branchiopoda**									
	Daphnia pulex	EFX70258.1	DappuP5A1	QGKLLRTILF	PPELPIE	FSSALKR	KGAPE	GDGTND	
**Insecta**									
	Drosophila melanogaster	NP_609490.1	DromeP5A1	QGKLLRTILF	PPDLPIE	FSSALKR	KGAPE	GDGTND	
**Cnidaria**									
**Anthozoa**									
	Nematostella vectensis	XP_001636788.1	NemveP5A1	QGKLLRTILF	PPELPIE	FSSALKR	KGAPE	GDGTND	
**Porifera**									
	Amphimedon queenslandica	XP_011403965.1	AmpquP5A1	QGKLLRTMLF	PPELPIE	FSSSLKR	KGAPE	GDGTND	
**Placozoa**									
	Trichoplax adhaerens	XP_002109418.1	TriadP5A1	QGKLLRTILY	PPELPIE	FSSVLKR	KGAPE	GDGTND	
**Ichthyosporea**									
	Capsaspora owczarzaki ATCC 30864	XP_004348060.1	CapowP5A1	QGKLVRTILF	PPELPME	FSSALKR	KGAPE	GDGTND	
**Fungi**									
**Ascomycota**									
**Eurotiomycetes**									
	Aspergillus nidulans FGSC A4	XP_660750.1	AspniP5A1	QGSLVRTMIY	PPELPME	FSSALKR	KGAPE	GDGTND	
**Leotiomycetes**									
	Blumeria graminis f. sp. hordei DH14	CCU75326.1	BlugrP5A1	QGSLVRTMIY	PPELPME	FSSALKR	KGAPE	GDGTND	
**Sordariomycetes**									
	Fusarium oxysporum Fo47	EWZ38181.1	FusoxP5A1	QGNLVRTMIY	PPELPME	FSSALKR	KGAPE	GDGTND	
**Dothideomycetes**									
	Setosphaeria turcica Et28A	XP_008026118.1	SettuP5A1	QGSLVRTMIF	PPELPME	FSSALKR	KGAPE	GDGTND	Extended N-terminal sequence
**Pezizomycetes**									
	Pyronema omphalodes CBS 100304	CCX07594.1	PyromP5A1	QGSLVRTMIY	PPELPME	FSSALKR	KGAPE	GDGTND	
**Saccharomycetes**									
	Saccharomyces cerevisiae	P39986	SacceP5A1/Spf1p	QGSLVRVMIY	PPELPME	FSSALKR	KGAPE	GDGTND	
	Candida albicans GC75	KGR00258.1	CanalP5A1	QGSLVRMMIF	PPELPME	FSSALKR	KGAPE	GDGTND	
**Schizosaccharomycetes**									
	Schizosaccharomyces pombe 972h-	NP_593971.1	SchpoP5A1	QGSLVRTMVF	PSELPME	FSSALKR	KGAPE	GDGTND	
**Basidiomycota**									
**Agaricomycetes**									
	Agaricus bisporus var. burnettii JB137-S8	XP_007331520.1	AgabiP5A1	QGQLVRTMIF	PPELPME	FSSALKR	KGAPE	GDGTND	
	Fibroporia radiculosa	XP_012185485.1	FibraP5A1	QGQLVRTMIF	PPELPME	FSSALKR	KGAPE	GDGTND	
**Ustilaginomycetes**									
	Ustilago maydis 521	XP_011386260.1	UstmaP5A1	QGQLIRLMVF	PPELPME	FSSALKR	KGAPE	GDGTND	
	Pseudozyma antarctica T-34	GAC73608.1	PseanP5A1	QGQLIRLMVF	PPELPME	FSSALKR	KGAPE	GDGTND	
**Pucciniomycetes**									
	Puccinia graminis f. sp. tritici CRL 75-36-700-3	XP_003328624.2	PucgrP5A1	QGQLIRTMIF	PPELPME	FSSLLKR	KGAPE	GDGTND	
**Wallemiomycetes**									
	Wallemia sebi CBS 633.66	XP_006956143.1	WalseP5A1	QGQLIRTMLF	PPELPME	FSSALKR	KGAPE	GDGTND	
**Glomeromycota**									
**Glomeromycetes**									
	Rhizophagus irregularis	ERZ97351.1	RhiirP5A1	QGKLVRTMVY	PPELPME	FSSALKR	KGAPE	GDGTND	
**Zygomycota**									
**Mucoromycotina**									
	Mucor circinelloides f. circinelloides 1006PhL	EPB82074.1	MucciP5A1	QGELVRTMIF	PPELPME	FSSALKR	KGAPE	GDGTND	
**Chytridiomycota**									
**Chytridiomycetes**									
	Batrachochytrium dendrobatidis JAM81	XP_006680616.1	BatdeP5A1	QGKLVRTIVY	PPELPME	FSSSLKR	KGAPE	GDGTND	
**Viridiplantae**									
**Streptophyta**									
**Embryophyta**									
**Magnoliophyta**									
**Eudicotyledons**									
	Arabidopsis thaliana	Q9LT02	ArathP5A1/MIA	QGKLMRTILF	PPELPME	FASHLKR	KGAPE	GDGTND	
	Medicago truncatula	KEH35668.1	MedtrP5A1	QGKLMRTILF	PPELPME	FASHLKR	KGAPE	GDGTND	
	Nicotiana sylvestris	XP_009763607.1	NicsyP5A1	QGKLMRTILF	PPELPME	FASHLKR	KGAPE	GDGTND	
	Theobroma cacao	XP_007029274.1	ThecaP5A1	QGKLMRTILF	PPELPME	FASHLKR	KGAPE	GDGTND	
**Early-diverging eudicotyledons**									
	Nelumbo nucifera	XP_010245696.1	NelnuP5A1	QGKLMRTILF	PPELPME	FASHLKR	KGAPE	GDGTND	
**Monocotyledons**									
	Zea mays	XP_008656297.1	ZeamaP5A1	QGKLMRTILF	PPELPME	FASHLKR	KGAPE	GDGTND	
	Elaeis guineensis	XP_010938896.1	ElaguP5A1	QGKLMRTILF	PPELPME	FASHLKR	KGAPE	GDGTND	
	Phoenix dactylifera	XP_008793945.1	PhodaP5A1	QGKLMRTILF	PPELPME	FASHLKR	KGAPE	GDGTND	
	Musa acuminata subsp. malaccensis	XP_009420982.1	MusmaP5A1	QGKLMRTILF	PPELPME	FASHLKR	KGAPE	GDGTND	
**Basal Magnoliophyta**									
	Amborella trichopoda	XP_011621107.1	AmbtrP5A1	QGKLMRTILF	PPELPME	FASHLKR	KGAPE	GDGTND	
**Lycopodiopsida (club mosses)**									
	Selaginella moellendorffii	XP_002992998.1	SelmoP5A1	QGKLMRTILF	PPELPME	FSSSLKR	KGAPE	GDGTND	N-terminal part of sequence missing
**Bryophyta (mosses)**									
	Physcomitrella patens	XP_001753025.1	PhypaP5A1	QGKLMRTILF	PPELPME	FASHLKR	KGAPE	GDGTND	
**Klebsormidiophyceae**									
	Klebsormidium flaccidum	kfl00235_0200_v1.1	KlefIP5A1	QGKLMRTILF	PPELPME	FASHLKR	KGAPE	GDGTND	
**Chlorophyta**									
**Chlorophyceae**									
	Chlamydomonas reinhardtii	XP_001703596.1	ChlreP5A1	QGRLMRTILF	PPELPME	FSSHLKV	KGAPE	GDGTND	
	Volvox carteri f. nagariensis	XP_002957878.1	VolcaP5A1	QGRLMRTILF	PPELPME	FSSHLKR	KGAPE	GDGTND	
	Coccomyxa subellipsoidea C-169	XP_005649928.1	CocsuP5A1	QGQLMRTILY	PPELPME	FTSTLKR	KGAPE	GDGTND	N-terminal part of sequence missing
**Trebouxiophyceae**									
	Chlorella variabilis	XP_005850052.1	ChlvaP5A1	QGRLMRTILY	PPELPME	FNSVLKR	KGAPE	GDGTND	
	Auxenochlorella protothecoides	XP_011398630.1	AuxprP5A1	QGRLMRTILY	PPELPME	FSSTLKR	KGAPE	GDGTND	
**Mamiellophyceae**									
	Micromonas pusilla CCMP1545	XP_003060956.1	MicpuP5A1	QGKLMRMMEF	PPELPMQ	FSSALQR	KGSPE	GDGAND	N-terminal part of sequence missing
	Micromonas commoda	XP_002502840.1	MicCCP5A1	QGKLMRMMEF	PPELPMQ	FSSALQR	KGSPE	GDGAND	
	Ostreococcus tauri	CEG00124.1	OsttaP5A1	QGKLMRMMEF	PPDLPMQ	FASALQR	KGSPE	GDGAND	
	Ostreococcus lucimarinus CCE9901	XP_001421103.1	OstluP5A1	QGKLMRMMEF	PPDLPMQ	FASALQR	KGSPE	GDGAND	N-terminal part of sequence missing
	Bathycoccus prasinos	XP_007513495.1	BatprP5A1	QGKLMRMMEF	PPELPMQ	FASSLQR	KGSPE	GDGAND	
**Rhodophyta**									
**Florideophyceae**									
	Gracilariopsis chorda	PXF50115.1	GrachP5A1	QGKLMRTIMY	PPELPMQ	FASALQR	KGSPE	GDGTND	
	Chondrus crispus	XP_005713359.1	ChocrP5A1	QGKLMRTILY	PPELPMQ	FASKLQR	KGSPE	GDGTND	N-terminal part of sequence missing
**Bangiophyceae**									
	Porphyra umbilicalis	OSX81029.1	PorumP5A1	QGRLMRTILF	PPELPMQ	FSSALQR	KGSPE	GDGTND	

			Galdieria sulphuraria	XP_005702263.1	GalsuP5A1	QGKLMRTILL	PPELPMQ	FSSALQR	KGSPE	GDGTND	N-terminal part of sequence missing
			Porphyridium purpureum	evm.model.contig_2085.12	PorpuP5A1	QGKLMRTIMY	PPELPMQ	FLSALQR	KGSPE	GDGTND	
			Cyanidioschyzon merolae strain 10D	XP_005537131.1	CyameP5A1	QGKLLRTIMY	PPELPMQ	FDATIQR	KGSPE	GDGTND	
**SAR**											
	**Stramenopiles**										
		**Oomycetes**									
			Phytophthora sojae	XP_009536938.1	PhysoP5A1	QGSLMRTILY	PPELPME	FSSELKR	KGAPE	GDGTND	
				XP_009519683.1	PhysoP5A2	QGKLVRMIEF	PAELPMQ	FASKLQR	KGSPE	GDGGND	
			Aphanomyces astaci	XP_009824842.1	AphasP5A1	QGNLMRTILF	PPELPME	FSSELKR	KGAPE	GDGTND	
				XP_009825846.1	AphasP5A2	QGKLVRMIEY	PAELPMQ	FASKLQR	KGSPE	GDGGND	
			Saprolegnia diclina VS20	XP_008604219.1	SapdiP5A1	QGNMLRTILL	PPELPME	FSSELRR	KGAPE	GDGTND	
				XP_008617435.1	SapdiP5A2	QGKLVRMIEF	PAELPMQ	FASKLQR	KGSPE	GDGAND	
			Albugo laibachii Nc14	CCA14384.1	AlblaP5A1	KGSLMRTILY	PPELPME	FSSELKR	KGAPE	GDGTND	
				CCA15951.1	AlblaP5A2	QGKLLRMIEF	PAELPMQ	FASKLQR	KGSPE	GDGGND	
		**Bacillariophyta**									
			Fragilariopsis cylindrus CCMP 1102	jgi|Fracy1|262228	FracyP5A1	QGSLLRTMAH	PPELPME	FSSRLKR	KGAPE	GDGTND	
				jgi|Fracy1|206011	FracyP5A2	QGKLVRMIEG	PPELPMQ	FSSKLQR	KGSPE	GDGAND	N-terminal part of sequence missing
			Pseudo-nitzschia multiseries CLN-47	jgi|Psemu1|246134	PsemuP5A1	QGSLLRTMAH	PPELPME	FSSKLKR	KGAPE	GDGTND	
				jgi|Psemu1|233650	PsemuP5A2	QGKLVRMIEG	PPELPMQ	FSSKLQR	KGSPE	GDGAND	N-terminal part of sequence missing
			Thalassiosira pseudonana CCMP1335	XP_002292624.1	ThapsP5A1	QGQLLRTMAY	PPELPME	FSSKLRR	KGAPE	GDGTND	N-terminal part of sequence missing
				XP_002295999.1	ThapsP5A2	QGKLVRMIEG	PPELPMQ	FSSKLQR	KGSPE	GDGAND	N-terminal part of sequence missing
			Phaeodactylum tricornutum CCAP 1055/1	XP_002182337.1	PhatrP5A1	QGSLLRTMAH	PPELPME	FSSKLKR	KGAPE	GDGTND	N-terminal part of sequence missing
		**Phaeophyceae**									
			Ectocarpus siliculosus	CBN79742.1	EctsiP5A1	QGQLMKTILF	PPELPIE	FDPSLRR	KGAPE	GDGTND	
				CBJ49156.1	EctsiP5A2	QGKLVRMIEG	PPELPMQ	FSSKLQR	KGSPE	GDGAND	
		**Labyrinthulomycetes**									
			Aplanochytrium kerguelense	jgi|Aplke1|54274	AplkeP5A1	QGSLVRTILF	PPELPME	FSSALKR	KGAPE	GDGTND	
				jgi|Aplke1|45929	AplkeP5A2	QGELMQMIEF	PQTLPVQ	FASKLQR	KGSPE	GDGGND	
			Aurantiochytrium limacinum ATCC MYA-1381	jgi|Aurli1|112332	AurliP5A1	QGSLMRTILF	PPELPME	FSSTLKR	KGAPE	GDGTND	
				jgi|Aurli1|42810	AurliP5A2	QGELMQMIEF	PRQLPVQ	FSSKLQR	KGSPE	GDGGND	
			Schizochytrium aggregatum ATCC 28209	jgi|Schag1|64641	SchagP5A1	–	PPELPME	FSSEAKR	KGAPE	GDGTND	Partial sequence from A domain missing
				jgi|Schag1|41186	SchagP5A2	QGELMQMIEF	PRQLPIQ	FSSKLQR	KGSPE	GDGGND	N-terminal part of sequence missing
	**Alveolata**										
		**Chromerida**									
			Vitrella brassicaformis CCMP3155	CEM01515.1	VitbrP5A1	QGKLVRTILF	PPEFPIT	FLSENQR	KGSAE	GDGTND	
		**Ciliophora**									
			Paramecium tetraurelia strain d4-2	XP_001439867.1	ParteP5A1	KGKLIKTVFY	PPELPMI	FKSDLKR	KGAPE	GDGTND	
			Pseudocohnilembus persalinus	KRW98783.1	PsepeP5A1	KGKLIRTVLH	PPELPMI	FRSDLKR	KGAPE	GDGTND	
			Tetrahymena thermophila SB210	XP_001025603.2	TetthP5A1	KGKLIRTVIF	PPELPMI	FKSDLKR	KGAPE	GDGTND	
			Stylonychia lemnae	CDW77809.1	StyleP5A1	QGKLMRTILF	PPELPMQ	FESALKR	KGAPE	GDGTND	
			Oxytricha trifallax	EJY76239.1	OxytrP5A1	QGKLMRTILF	PPELPMQ	FESALKR	KGAPE	GDGTND	
			Ichthyophthirius multifiliis	XP_004030454.1	IchmuP5A1	KGKLIRTVLH	PPELPMI	FRSDLKR	KGAPE	GDGTND	
		**Perkinsida**									
			Perkinsus marinus ATCC 50983	XP_002765522.1	PermaP5A1	QGKLVRTILY	PPEFPVT	FNSELQR	KGSPE	GDGTND	
				XP_002769871.1	PermaP5A2	QGELTRMIEF	PPELPMQ	FSSALQR	KGSPE	GDGGND	
		**Apicomplexa**									
			Toxoplasma gondii ME49	XP_002369785.1	ToxgoP5A1	QGKLVRTILF	PAEFPIT	FSSALQR	KGSPE	GDGTND	
			Babesia microti strain RI	XP_012649572.1	BabmiP5A1	QGKLVKSLLH	PAEFPIT	FASHLQR	KGSPE	GDGTND	
			Plasmodium knowlesi strain H	XP_002257796.1	PlaknP5A1	QGKLVRTIIN	PPEFPIT	FSSELQR	KGSPE	GDGTND	
			Gregarina niphandrodes	XP_011130755.1	GreniP5A1	QGKLIRTVLY	PPELPIT	FASEFRR	KGAPE	GDGTND	
			Hammondia hammondi	XP_008885252.1	HamhaP5A1	QGKLVRTILF	PAEFPIT	FSSALQR	KGSPE	GDGTND	
			Neospora caninum Liverpool	CEL65253.1	NeocaP5A1	QGKLVRTILY	PAEFPIT	FSSVLQR	KGSPE	GDGTND	
	**Rhizaria**										
		**Cercozoa**									
			Plasmodiophora brassicae	CEO98872.1	PlabrP5A1	QGRLLSTILC	PPELPME	FESALRR	KGAPE	GDGTND	
			Bigelowiella natans CCMP2755	jgi|Bigna1|41530	BignaP5A1	QGNLVRTILF	PPELPME	FSSELQR	KGSPE	GDGTND	N-terminal part of sequence missing
				jgi|Bigna1|86482	BignaP5A2	QGELMQMIEF	PRQLPMQ	FLSKLQR	KGSPE	GDGGND	
	**Cryptophyta**										
		**Pyrenomonadales**									
			Guillardia theta CCMP2712	XP_005832391.1	GuithP5A1	QGRLVRTIMF	PPELPMN	FASSLKR	KGAPE	GDGTND	N-terminal part of sequence missing
				XP_005835651.1	GuithP5A2	EGKLVRMIEY	PPELPMQ	FASKLQR	KGSPE	GDGAND	
	**Haptophyta**										
		**Isochrysidales**									
			Emiliania huxleyi CCMP1516	XP_005791913.1	EmihuP5A1	QGELMQMIEF	PRHLPMQ	FSSALQR	KGSPE	GDGGND	
		**Prymnesiales**									
			Chrysochromulina sp. CCMP291	KOO24184.1	ChrCCP5A1	QGELMRMIEF	PPELPMQ	FASSLGR	KGSPE	GDGGND	
**Amoebozoa**											
		**Mycetozoa**									
			Dictyostelium discoideum AX4	XP_629288.1	DicdiP5A1	QGRLMRTIWF	PPELPME	FSSELKR	KGAPE	GDGTND	
			Polysphondylium pallidum PN500	EFA80291.1	ParpaP5A1	QGSLMRTIWF	PPELPME	FSSDLKR	KGAPE	GDGTND	
			Acytostelium subglobosum LB1	GAM22379.1	AcysuP5A1	QGSLMRTIWF	PPELPME	FSSDLKR	KGAPE	GDGTND	
**Discobids**											
		**Euglenozoa**									
			Strigomonas culicis	EPY18510.1	StrcuP5A1	QGKLLRTILH	PSELPME	FLAALRR	KGSPE	GDGTND	
			Trypanosoma cruzi	XP_814797.1	TrycrP5A1	QGKLLRTILH	PPELPME	FSANLRR	KGSPE	GDGTND	
			Leishmania major strain Friedlin	XP_001681016.1	LeimaP5A1	QGKLLRTILH	PPELPME	FLATLRR	KGSPE	GDGTND	

Table S3

EF2-like proteins in selected organisms

Taxonomy	*Species*	*Acc. Nr.*	*Abbrev. name*
**Eukaryota**			
**Opisthokonta**			
**Metazoa**			
**Deuterostomia**			
**Chordata**			
**Mammalia**			
	Homo sapiens	Q7Z2Z2	HomsaEF2L
		P13639	HomsaEF2
		Q15029	HomsaEFTU
		Q96RP9	HomsaEF-Gm
**Sarcopterygii (lobe-finned fish)**			
	Latimeria chalumnae	XP_005999104.1	LatchEF21
		XP_006008618.1	LatchEF22
		XP_006000383.1	LatchEFTU
		XP_006002223.1	LatchEF-Gm
**Tunicata**			
	Ciona intestinalis	XP_009861858.1	CioinEF2L
		XP_009858621.1	CioinEF21
		XP_009858635.1	CioinEF22
		XP_002129683.1	CioinEFTU
		XP_002128337.1	CuioinEF-Gm
**Protostomia**			
**Brachiopoda**			
**Lingulata**			
	Lingula anatina	XP_013413557.1	LinanEF2L
		XP_013394736.1	LinanEF2
		XP_013419341.1	LinanEFTU
		XP_013401292.1	LinanEF-Gm
**Arthropoda**			
	Drosophila melanogaster	NP_788515.1	DromeEF2L
		NP_525105.2	DromeEF2
		NP_651605.1	DromeEFTU
		NP_609105.1	DromeEF-Gm
**Nematoda**			
**Chromadorea**			
	Caenorhabditis elegans	NP_001251010.1	CaeelEF2
		NP_498308.1	CaeelEFTU
		NP_496787.1	CaeelEF-Gm
**Placozoa**			
	Trichoplax adhaerens	XP_002116427.1	TriadEF2L
		XP_002110547.1	TriadEF2
		XP_002111284.1	TriadEFTU
		XP_002116194.1	TriadEF-Gm
**Fungi**			
**Ascomycota**			
**Saccharomycetes**			
	Saccharomyces cerevisiae	P53893	SacceEF2L/Ria1p
		P32324	SacceEF2/Eft1p
		NP_012748.1	SacceEFTU/Snu114p
		P25039	SacceEF-Gm/Mef1p
**Schizosaccharomycetes**			
	Schizosaccharomyces pombe 972h-	NP_587766.1	SchpoEF2L
**Basidiomycota**			
**Agaricomycetes**			
	Agaricus bisporus var. burnettii H97	XP_006462620.1	AgabiEF2L
		XP_006454257.1	AgabiEF2
		XP_006453987.1	AgabiEFTU
		XP_006456450.1	AgabiEF-Gm
	Ustilago maydis 521	XP_011387475.1	UstmaEF2L
		XP_011390785.1	UstmaEF2
		XP_011389303.1	UstmaEFTU
**Glomeromycota**			
**Glomeromycetes**			
	Rhizophagus irregularis DAOM 181602	ERZ96264.1	RhiirEF2L
		ESA05806.1	RhiirEF21
		ESA13708.1	RhiirEF22
		ESA15126.1	RhiirEF-Gm
**Zygomycota**			
**Mucoromycotina**			
	Rhizopus delemar RA 99-880	EIE76664.1	RhideEF2L
		EIE84099.1	RhideEF2
		EIE82824.1	RhideEF-Gm
**Chytridiomycota**			
**Chytridiomycetes**			
	Spizellomyces punctatus DAOM BR117	KNC99015.1	SpipuEF2L
		KNC96507.1	SpipuEF2
		KND01710.1	SpipuEFTU
		KND00418.1	SpipuEF-Gm
**Cryptomycota**			
	Rozella allomycis CSF55		
		EPZ31247.1	RozalEF2L
		EPZ34723.1	RozalEF2
		EPZ31223.1	RozalEF-Gm
**Viridiplantae**			
**Streptophyta**			
**Embryophyta**			
**Magnoliophyta**			
**Eudicotyledons**			
	Arabidopsis thaliana	Q9LS91	ArathEF2L
		Q9ASR1	ArathEF21
		F4JB05	ArathEF22
		Q9LNC5	ArathEFTU1
		F4JWP9	ArathEFTU2
		Q9SI75	ArathEF-Gp
		Q9C641	ArathEF-Gm_1
		F4IW10	ArathEF-Gm_2
**Klebsormidiophyceae**			
	Klebsormidium flaccidum	kfl00142_0070	KleflEF2L
		kfl00080_0090	KleflEF2
		kfl00373_0110	KleflEFTU
		kfl00020m_0070	KleflEF-Gm
		kfl00160_0040	KleflEF-Gp
**Chlorophyta**			
**Chlorophyceae**			
	Chlamydomonas reinhardtii	XP_001703215.1	ChlreEF2
		XP_001699617.1	ChlreEFTU
		XP_001701845.1	ChlreEF-Gp
	Volvox carteri f. nagariensis	XP_002947572.1	VolcaEF2L
		XP_002947914.1	VolcaEF2
		XP_002947666.1	VolcaEFTU
		XP_002956194.1	VolcaEF-Gp
	Coccomyxa subellipsoidea C-169	XP_005649395.1	CocsuEF2L
		XP_005646726.1	CocsuEF2
		XP_005648748.1	CocsuEFTU
		XP_005649220.1	CocsuEF-Gm
		XP_005644650.1	CocsuEF-Gp
**Trebouxiophyceae**			
	Chlorella variabilis	XP_005852008.1	ChlvaEF-Gm
	Parachlorella kessleri	P28996	ParkeEF2
	Auxenochlorella protothecoides	XP_011398157.1	AuxprEF2
		XP_011398222.1	AuxprEF-Gm
		XP_011402389.1	AuxprEF-Gp
	Helicosporidium sp. ATCC 50920	KDD76285.1	HelATEF-Gm
**Mamiellophyceae**			
	Micromonas pusilla CCMP1545	XP_003063602.1	MicpuEF2L
		XP_003059677.1	MicpuEF2

				XP_003058292.1	MicpuEFTU
				XP_003057858.1	MicpuEF-Gm
				XP_003057849.1	MicpuEF-Gp
			Micromonas commoda	XP_002504618.1	MicRCEF2L
				XP_002505405.1	MicRCEF2
				XP_002499530.1	MicRCEFTU
				XP_002500070.1	MicRCEF-Gm
				XP_002500081.1	MicRCEF-Gp
			Ostreococcus tauri	CEG00872.1	OsttaEF2L
				CEG02074.1	OsttaEF2
				XP_003080142.1	OsttaEFTU
				XP_003080490.1	OsttaEF-Gm
				XP_003080548.1	OsttaEF-Gp
			Ostreococcus lucimarinus CCE9901	XP_001422442.1	OstluEF2L
				XP_001422701.1	OstluEF2
				XP_001418820.1	OstluEFTU
				XP_001418768.1	OstluEF-Gm
			Bathycoccus prasinos	XP_007511672.1	BatprEF2L
				XP_007509777.1	BatprEF2
				XP_007508471.1	BatprEFTU
				XP_007513725.1	BatprEF-Gm
				XP_007508434.1	BatprEF-Gp
**Rhodophyta**					
		**Florideophyceae**			
			Chondrus crispus	XP_005717389.1	ChocrEF2
				XP_005710237.1	ChocrEFTU
				XP_005711388.1	ChocrEF-Gm
				XP_005719168.1	ChocrEF-Gp
		**Bangiophyceae**			
			Cyanidioschyzon merolae strain 10D	XP_005536276.1	CyameEF2L
				XP_005539022.1	CyameEF2
				XP_005535977.1	CyameEF-Gm
				XP_005536561.1	CyameEF-Gp
			Galdieria sulphuraria	XP_005702526.1	GalsuEF2L
				XP_005703879.1	GalsuEF2
				XP_005703436.1	GalsuEFTU
				XP_005708926.1	GalsuEF-Gm
				XP_005705782.1	GalsuEF-Gp
**Glaucocystophyceae**					
		**Cyanophoraceae**			
			Cyanophora paradoxa	Contig7966-abinit-gene-0.4	CyapaEF2
**SAR**					
	**Stramenopiles**				
		**Oomycetes**			
			Phytophthora parasitica INRA-310	XP_008897851.1	PhypaEF2L
				XP_008891756.1	PhyparEF2
				ETK85775.1	PhypaEFTU
				XP_008898647.1	PhypaEF-Gm
			Aphanomyces astaci	XP_009823253.1	AphasEF2L
				XP_009825782.1	AphasEF2
				XP_009822108.1	AphasEFTU
				XP_009830277.1	AphasEF-Gm
			Saprolegnia diclina VS20	XP_008604581.1	SapdiEFTU
				XP_008605882.1	SapdiEF-Gm
			Albugo laibachii Nc14	CCA26090.1	AlblaEF2L
				CCA22142.1	AlblaEF-Gm
			Albugo candida	CCI11527.1	AlbcaEF2L
				CCI49602.1	AlbcaEFTU
			Plasmopara halstedii	CEG35619.1	PlahaEF2L
				CEG40323.1	PlahaEFTU
				CEG37279.1	PlahaEF-Gm
		**Bacillariophyta**			
			Thalassiosira pseudonana CCMP1335	XP_002291136.1	ThapsEF2L
				XP_002291169.1	ThapsEF2
				XP_002286996.1	ThapsEFTU
				XP_002294597.1	ThapsEF-Gp
			Thalassiosira oceanica CCMP1005	EJK57124.1	ThaocEF2L
				EJK52971.1	ThaopcEF-G
			Phaeodactylum tricornutum CCAP 1055/1	XP_002180100.1	PhatrEF2
				XP_002179302.1	PhatrEFTU
				XP_002178766.1	PhatrEF-Gp
		**Eustigmatophyceae**			
			Nannochloropsis gaditana	EWM29754.1	NangaEF2L
				EWM28125.1	NangaEFTU
				EWM26600.1	NangaEF-Gp
		**Pelagophyceae**			
			Aureococcus anophagefferens	XP_009041316.1	AuranEF2
				XP_009033668.1	AuranEFTU
				XP_009035581.1	AuranEF-Gm
		**Phaeophyceae**			
			Ectocarpus siliculosus	CBJ26987.1	EctsiEF2L
				CBJ32863.1	EctsiEF2
				CBN76883.1	EctsiEFTU
				CBN78883.1	EctsiEF-Gp
		**Labyrinthulomycetes**			
			Aplanochytrium kerguelense	jgi|Aplke1|83213	AplkeEF2L
				jgi|Aplke1|91534	AplkeEF2
			Aurantiochytrium limacinum ATCC MYA-1381	jgi|Aurli1|37878	AurliEF2L
				jgi|Aurli1|125465	AurliEF2
			Schizochytrium aggregatum ATCC 28209	jgi|Schag1|39285	SchagEF2L
				jgi|Schag1|83215	SchagEF2
		**Blastocystis**			
			Blastocystis hominis	XP_012897160.1	BlahoEF21
				Q17152.1	BlahoEF22
				XP_012894082.1	BlahoEF-Gm
	**Alveolata**				
		**Chromerida**			
			Vitrella brassicaformis CCMP3155	CEL94296.1	VitbrEF2L
				CEM25456.1	VitbrEF21
				CEL92355.1	VitbrEF22
				CEM07887.1	VitbrEFTU
				CEM31496.1	VitbrEF-G
				CEM00559.1	VitbrEF-Gm
		**Ciliophora**			
			Paramecium tetraurelia strain d4-2	XP_001458816.1	ParteEF2L
				XP_001433655.1	ParteEF21
				XP_001435182.1	ParteEF22
				XP_001430017.1	ParteEFTU
				XP_001434958.1	ParteEF-Gm
			Tetrahymena thermophila SB210	XP_001018683.2	TettheEF2L
				XP_001010970.1	TetthEF21
				XP_001020227.1	TetthEF22
				XP_001031057.2	TetthEF23
				XP_001015831.1	TetthEFTU
				XP_001029970.2	TetthEF-Gm
			Stylonychia lemnae	CDW88668.1	StyleEF2L
				CDW85404.1	StyleEF21
				CDW80370.1	StyleEF22
				CDW87448.1	StyleEFTU
				CDW80931.1	StyleEF-Gm
			Oxytricha trifallax	EJY75186.1	OxytrEF2L
				EJY87908.1	OxytrEFTU
				EJY65330.1	OxytrEF-Gm
			Ichthyophthirius multifiliis	XP_004031079.1	IchmuEF2L
		**Perkinsida**			
			Perkinsus marinus ATCC 50983	XP_002786006.1	PermaEF2
				XP_002784120.1	PermaEF-Gm
		**Apicomplexa**			

			Toxoplasma gondii ME49	XP_002367778.1	ToxgoEF2
				XP_002369245.1	ToxgoEFTU
				XP_002365190.1	ToxgoEF-Gm
				XP_002366083.1	ToxgoEF-G
			Babesia microti strain RI	XP_012649502.1	BabmiEF2
				XP_012647168.1	BabmiEFTU
				XP_012647939.1	BabmiEF-G
			Plasmodium falciparum 3D7	XP_001348660.1	PlafaEF2
				XP_001350724.1	PlafaEF-G
			Theileria equi	XP_004833795.1	TheeqEF2L
				XP_004830685.1	TheeqEFTU
			Cryptosporidium parvum	Q23716.1	CrypaEF2
				XP_626934.1	CrypaEFTU
				XP_001388343.1	CrypaEF2L
			Cryptosporidium hominis	CUV06821.1	CryhoEF2L
			Hammondia hammondi	XP_008887474.1	HamhaEF2
				XP_008884138.1	HamhaEFTU
				XP_008883029.1	HamhaEF-Gm
			Eimeria tenella	XP_013227887.1	EimteEFTU
				XP_013232186.1	EimteEF-Gm
	**Rhizaria**				
		**Cercozoa**			
			Plasmodiophora brassicae	CEO96359.1	PlabrEF2
				CEP00817.1	PlabrEFTU
				CEO96132.1	PlabrEF2L
				CEO98361.1	PlabrEF-Gm
			Amorphochlora amoebiformis	BAS01792.1	AmoamEF2
			Lotharella oceanica	AIB09773.1	LotocEF2
			Paulinella chromatophora	YP_002049196.1	PauchEF-G
			Bigelowiella natans	XP_001712988.1	BignaEF2
	**Cryptophyta**				
		**Pyrenomonadales**			
			Guillardia theta CCMP2712	XP_001713413.1	GuithEF21
				XP_005833034.1	GuithEF22
				XP_005838745.1	GuithEF-Gp
			Chroomonas mesostigmatica CCMP1168	AFP65695.1	ChrmeEF2
		**Cryptomonodales**			
			Hemiselmis andersenii	XP_001712192.1	HemanEF2
			Cryptomonas paramecium	XP_003239701.1	CryparEF2
	**Haptophyta**				
		**Isochrysidales**			
			Emiliania huxleyi CCMP1516	XP_005776251.1	EmihuEF2L
				XP_005763155.1	EmihuEFTU
				XP_005790815.1	EmihuEF-Gm
		**Prymnesiales**			
			Chrysochromulina sp. CCMP291	KOO21605.1	ChrCCEFTU
				KOO30316.1	ChrCCEF-Gm
				KOO21676.1	ChrCCEF-Gp
**Amoebozoa**					
		**Archamoebae**			
			Entamoeba histolytica HM-1:IMSS	XP_651009.2	EnthiEF2
				XP_656735.2	EnthiEFTU
				XP_655775.1	EnthiEF2L
**Discobids**					
		**Euglenozoa**			
			Trypanosoma cruzi strain CL Brener	XP_809041.1	TrycrEF2
				XP_812451.1	TrycrEFTU
				XP_805852.1	TrycrEF2L
				XP_806568.1	TrycrEF_Gm
		**Metamonada**			
			Giardia lamblia ATCC 50803	XP_001704820.1	GialaEF2
				XP_001707287.1	GialaEF2L
**Archaea**					
	**Euryarchaeota**				
		**Methanobacteria**			
			Methanococcoides methylutens	WP_048195088.1	MetmeEF1
				WP_048205754.1	MetmeEF2
			Methanocella conradii	WP_014406721.1	MetcoEF
			Methanosarcina thermophila	WP_048166885.1	MetthEF
			Methanosarcina barkeri	WP_048107408.1	MetbaEF
			Methanosarcina horonobensis	WP_048137973.1	MethoEF
			Methanoregula formicica	WP_015286777.1	MetfoEF
			Methanospirillum hungatei	WP_011449774.1	MethuEF
			Candidatus Methanoperedens nitroreducens	WP_048088503.1	MetniEF1
				WP_096203651.1	MetniEF2
			Methanosphaerula palustris	WP_012616981.1	MetpaEF
			Methanoregula boonei	WP_011991273.1	MetboEF
			Methanobacterium paludis	AEG17284.1	MetpalEF
		**Thermoplasmata**			
			Picrophilus torridus DSM 9790	AAT43002.1	PictoEF
			Thermoplasma acidophilum	P26752	TheacEF2
		**Halobacteria**			
			Haloterrigena limicola	WP_008008726.1	HalliEF
			Halobacterium salinarum	Q9HM85	HalsaEF2
			Natrinema pallidum	WP_006183995.1	NatpaEF
		**Methanococci**			
			Methanocaldococcus bathoardescens	WP_048201892.1	MetbatEF
			Methanococcus vannielii	P09604	MetvaEF2
		**Thermococci**			
			Pyrococcus yayanosii	WP_013906024.1	PyryaEF
			Pyrococcus woesei	P61878	PyrwoEF2
	**Crenarchaeota**				
		**Thermoprotei**			
			Ignicoccus hospitalis	WP_012123523.1	IgnhoEF
			Pyrodictium delaneyi	WP_055409731.1	PyrdeEF
			Hyperthermus butylicus	WP_011822009.1	HypbuEF
			Pyrolobus fumarii	WP_014027322.1	PyrfuEF
			Candidatus Acidianus copahuensis	WP_048100036.1	AcicoEF
			Metallosphaera yellowstonensis	WP_009073492.1	MetyeEF
			Acidianus manzaensis	ARM75919.1	AcimaEF
			Thermofilum sp. ex4484_79	OYT31738.1	TheesEF
			Sulfolobus tokodaii	WP_052846316.1	SultoEF
			Sulfolobus acidocaldarius	P23112/WP_015385460.1	SulacEF2/SulacEF
			Sulfolobus solfataricus	P30925	SulsoEF2
			Desulfurococcus mobilis	P33159	DesmoEF2
			Desulfurococcus mucosus	WP_013562824.1	DesmuEF
